# Comparative Efficacy and Precision of Robot-Assisted vs. Conventional Total Knee Arthroplasty: A Systematic Review and Meta-Analysis of Randomized Controlled Trials

**DOI:** 10.3390/jcm14093249

**Published:** 2025-05-07

**Authors:** Ümit Mert, Moh’d Yazan Khasawneh, Maher Ghandour, Ahmad Al Zuabi, Klemens Horst, Frank Hildebrand, Bertil Bouillon, Mohamad Agha Mahmoud, Koroush Kabir

**Affiliations:** 1 Department of Orthopaedics and Trauma Surgery, Helios University Hospital, University Witten/Herdecke, 42283 Wuppertal, Germany; mohdyazan.khasawneh@helios-gesundheit.de (M.Y.K.); mghandourmd@gmail.com (M.G.); koroush.kabir@helios-gesundheit.de (K.K.); 2 Department of Orthopedics, Trauma and Reconstructive Surgery, RWTH Aachen University, 52056 Aachen, Germany; ahmad.alzuabi@hotmail.com (A.A.Z.); khorst@ukaachen.de (K.H.); fhildebrand@ukaachen.de (F.H.); m.agha.mahmoud@gmail.com (M.A.M.); 3 Department of Traumatology and Orthopedic Surgery, Cologne-Merheim Medical Center (CMMC), University Witten/Herdecke, 51109 Cologne, Germany; bouillonb@kliniken-koeln.de

**Keywords:** robot-assisted total knee arthroplasty, conventional total knee arthroplasty, knee society score, radiographic outcomes, robotic systems, meta-analysis

## Abstract

**Background/Objectives**: Total knee arthroplasty (TKA) is a common procedure for knee osteoarthritis. While conventional TKA (C-TKA) remains standard, robot-assisted TKA (RA-TKA) has been introduced to enhance implant positioning and clinical outcomes. However, its comparative benefits remain unclear. This systematic review and meta-analysis compared RA-TKA with C-TKA, examining the influence of robotic system, surgeon experience, and follow-up duration. **Methods**: A systematic search was conducted across the PubMed, Scopus, Web of Science, and Cochrane Library databases. Randomized controlled trials (RCTs) comparing RA-TKA with C-TKA were included. Outcomes were categorized into clinical, radiographic, and safety endpoints. Subgroup and meta-regression analyses explored factors influencing outcome variability, including robotic system, number of surgeons, and follow-up duration. **Results**: Twenty-five RCTs (5614 patients) were analyzed. RA-TKA showed modest improvements in clinical outcomes, such as KSS and VAS pain scores, but results varied across subgroups. RA-TKA demonstrated a significantly better flexion range of motion (ROM) in certain countries (e.g., Russia, MD = 10; 95%CI: 5.44, 14.56) and with specific robotic systems (e.g., NAVIO). No significant differences were found in OKS and HSS scores. Radiographic outcomes, including the HKA Angle, varied by robotic system, with NAVIO and YUANHUA showing better alignment than C-TKA. Complication rates were comparable, though RA-TKA had a higher risk of conversion to open surgery (10% vs. 2%). Meta-regression identified robotic system and surgeon experience as key predictors of outcome variability. **Conclusions**: RA-TKA offers advantages in implant alignment and postoperative pain reduction. However, benefits are inconsistent across settings, and some robotic systems may not provide improvements over C-TKA.

## 1. Introduction

Total knee arthroplasty (TKA) is one of the most successful and frequently performed surgical procedures worldwide, primarily used to alleviate pain and improve function in patients suffering from end-stage knee osteoarthritis [1,2]. Globally, the number of TKAs performed has increased significantly, with over 1 million procedures conducted annually in the United States alone—a number projected to exceed 3 million by 2040 [3]. The rising incidence is largely driven by an aging population, increasing obesity rates, and the growing prevalence of osteoarthritis. Despite its success, traditional TKA approaches face limitations, particularly in achieving optimal alignment [4], balancing soft tissues [5], and ensuring long-term implant survival [6]. Moreover, TKA imposes a substantial economic burden on healthcare systems due to the high costs of surgery, rehabilitation, and potential revision procedures. Therefore, improving surgical precision and long-term outcomes remains a clinical priority. Malalignment, imprecision in component positioning, and soft tissue imbalance have been linked to suboptimal outcomes, including persistent pain, poor functional recovery, and the need for revision surgery [7].

In recent years, robot-assisted TKA (RA-TKA) has emerged as a promising innovation aimed at addressing these limitations [8]. Robotic systems aim to assist surgeons in achieving greater precision through advanced imaging and intraoperative feedback, potentially improving bone cuts, alignment, and soft tissue balance [9]. As a result, RA-TKA has been hypothesized to enhance clinical outcomes, increase patient satisfaction, and reduce the risk of complications associated with implant malpositioning [10,11]. Several robotic systems, such as MAKO [12], NAVIO [13], and ROBODOC^®^ [14], have been developed, each offering unique features intended to improve the accuracy and reproducibility of the procedure. Proper implant alignment and soft tissue balancing are key determinants of prosthesis longevity and patient satisfaction following TKA. Malalignment can increase the risk of implant loosening, pain, and functional limitations, leading to early revision [7]. RA-TKA systems aim to enhance surgical precision, potentially translating into improved long-term outcomes—particularly in patient populations with complex deformities, high BMI, or young age, where implant positioning and soft tissue management are more challenging and critical to function.

While early studies have reported promising outcomes with RA-TKA, the clinical and radiographic advantages of this technology over conventional TKA (C-TKA) remain a subject of debate [7,10,11,15,16,17]. Some studies have suggested that RA-TKA provides superior implant alignment and improved early functional outcomes, while others have reported no significant differences between the two techniques. Moreover, the variability in robotic systems, surgeon experience, and patient characteristics may influence the outcomes of RA-TKA, complicating the interpretation of results across studies.

Given the rising utilization of robotic systems in TKA and their associated financial implications [9], a comprehensive assessment is needed to determine whether specific robotic platforms offer clinically meaningful advantages in implant alignment, functional outcomes, or complication rates, particularly when adjusted for variables such as surgeon experience and follow-up duration.

Although several systematic reviews [18,19] have evaluated robot-assisted versus conventional TKA, these analyses were limited by smaller sample sizes, minimal subgroup exploration, or outdated literature coverage. Unlike prior reviews, we conducted subgroup and meta-regression analyses based on robotic system type, surgeon experience, and follow-up duration to uncover effect modifiers that may explain the heterogeneity in clinical and radiographic outcomes.

## 2. Materials and Methods

### 2.1. Design and Literature Search

This systematic review and meta-analysis followed the PRISMA [20] and AMSTAR [21] guidelines (protocol: CRD42024603379). We searched PubMed, Scopus, Web of Science, Cochrane Library, and Google Scholar (first 200 citations) up to 1 October 2024 [22]. The search strategy (detailed in Table S1) was adapted per database. No language restrictions were applied; three studies were translated from Chinese [23,24,25]. Reference lists and related articles were manually screened [26].

### 2.2. Selection Strategy

Studies were selected using the PICOS framework [27].

The inclusion criteria were as follows:

1. Population: patients with knee osteoarthritis.
2. Intervention: RA-TKA
3. Comparison: C-TKA.
4. Outcome: efficacy (clinical and radiographic) and safety endpoints. A full list is provided below.
5. Study Design: only randomized controlled trials (RCTs).

The exclusion criteria included the following:

1. Non-original research.
2. Abstract-only publications.
3. Non-randomized studies.
4. Duplicated records or studies with overlapping datasets.
5. Non-comparative studies or comparisons with non-TKA modalities.

### 2.3. Data Collection and Outcomes

The senior author designed the data collection sheet using Microsoft Excel. The final sheet comprised 3 parts. The first part covered study-related data (authors’ names, year of publication/investigation, country of investigation, study design, sample size, and follow-up period). The second part covered patient-related (age and gender) and intervention-related data (i.e., robotic system and surgeon experience). The third part included the outcome data, divided into clinical and radiographic parameters. Clinical parameters included Knee Society Score (KSS), Oxford Knee Score (OKS) pain using a Visual Analogue Scale (VAS), range of motion (ROM) during flexion and extension, Hospital for Special Surgery (HSS) score, operative time, intraoperative blood loss, satisfaction rate, and complications. Radiographic parameters included Hip–Knee–Ankle Angle, HKA Deviation, Femoral Coronal Inclination Angle, Tibial Coronal Inclination Angle, Femoral Sagittal Inclination Angle, Tibial Sagittal Inclination Angle, Transverse Femoral Angle, and Transverse Tibial Angle.

### 2.4. Risk of Bias Assessment

Assessment of the risk of bias (RoB) of the included RCTs was performed using the revised Cochrane’s RoB-2 tool, which assessed the methodology of each trial over 5 domains: randomization, deviation from intended interventions, missing outcome data, outcome measurement, and selective reporting. An overall rating of low, some concerns, or high risk was given [28].

### 2.5. Statistical Analysis

Analyses were performed in STATA—version 18 (StataCorp, College Station, TX, USA) using the “metan” and “metareg” commands. Continuous outcomes were reported as mean differences (MDs) with 95% confidence intervals (CIs), while dichotomous outcomes were expressed as odds ratios (ORs) with 95%CIs. Due to study heterogeneity, subgroup meta-analyses were conducted instead of a pooled analysis. A random-effects model (REML) was used, with I^2^ >75% indicating substantial heterogeneity. Cochran’s Q-test assessed significance.

Pre-specified subgroup analyses examined heterogeneity sources, stratified by country, risk of bias, surgeon experience, robotic system (e.g., MAKO and NAVIO), and follow-up duration. Meta-regression explored predictors of effect size, testing covariates such as mean age, gender ratio, robotic system, and study-level factors (sample size and follow-up duration). Multicollinearity was assessed using the variance inflation factor (VIF) [29], excluding highly collinear variables. R² values evaluated model fit, with *p* < 0.05 considered significant.

Publication bias was assessed via funnel plots and Egger’s test (>10 studies required). No asymmetry was observed. A Galbraith plot identified influential studies.

## 3. Results

### 3.1. Literature Search Results

**Figure 1:**
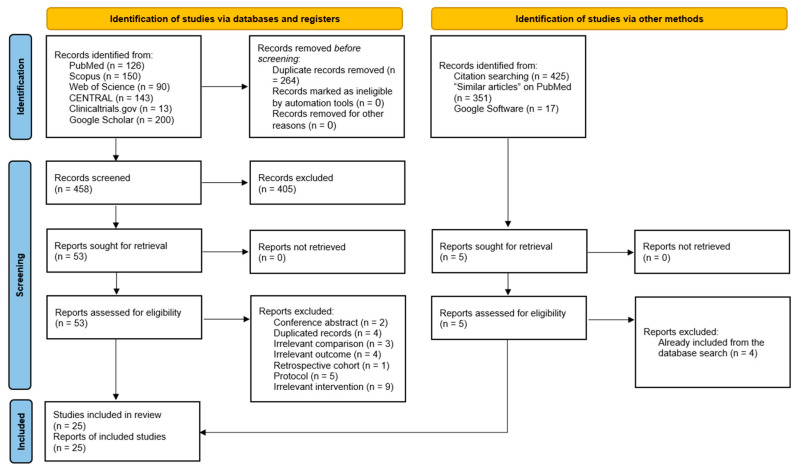
A PRISMA flow diagram showing the results of the systematic literature search.

The search identified 722 citations (Figure 1), with 264 duplicates removed. After screening 458 articles, 405 were excluded based on titles/abstracts. The remaining 53 full texts were assessed, leading to 28 exclusions (reasons given in Table S2). Ultimately, 25 RCTs were included in the analysis [23,24,25,30,31,32,33,34,35,36,37,38,39,40,41,42,43,44,45,46,47,48,49,50,51].

### 3.2. Baseline Characteristics of the Included Studies

Among the 25 included RCTs, only 9 had a registered protocol (Table 1). Most studies were conducted in China (n = 8) and South Korea (n = 6). A total of 5614 patients underwent TKA, with 2435 allocated to RA-TKA and 2109 to C-TKA. The most commonly used robotic system was ROBODOC (n = 8), followed by NAVIO (n = 4), MAKO (n = 3), and YUANHUA (n = 3). Follow-up ranged from 10 days to 10 years.

### 3.3. Risk of Bias Summary

**Figure 2:**
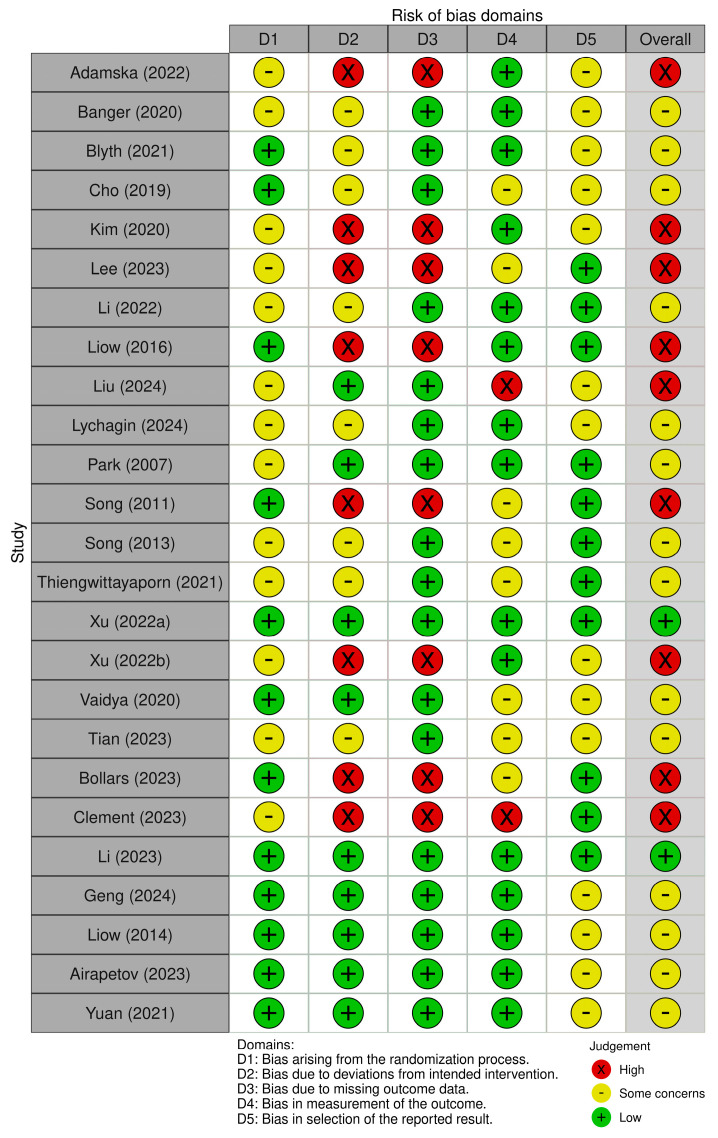
A graph showing the risk of bias summary of included randomized trials using the revised Cochrane risk of bias (ROB-2) tool.

Only two RCTs had a low risk of bias, while nine were rated as high-risk and fourteen as having some concerns. The primary methodological concerns included the absence of a registered protocol and unclear descriptions of randomization procedures (Figure 2).

### 3.4. Clinical Outcomes

#### 3.4.1. KSS Total and Functional Score

**Figure 3:**
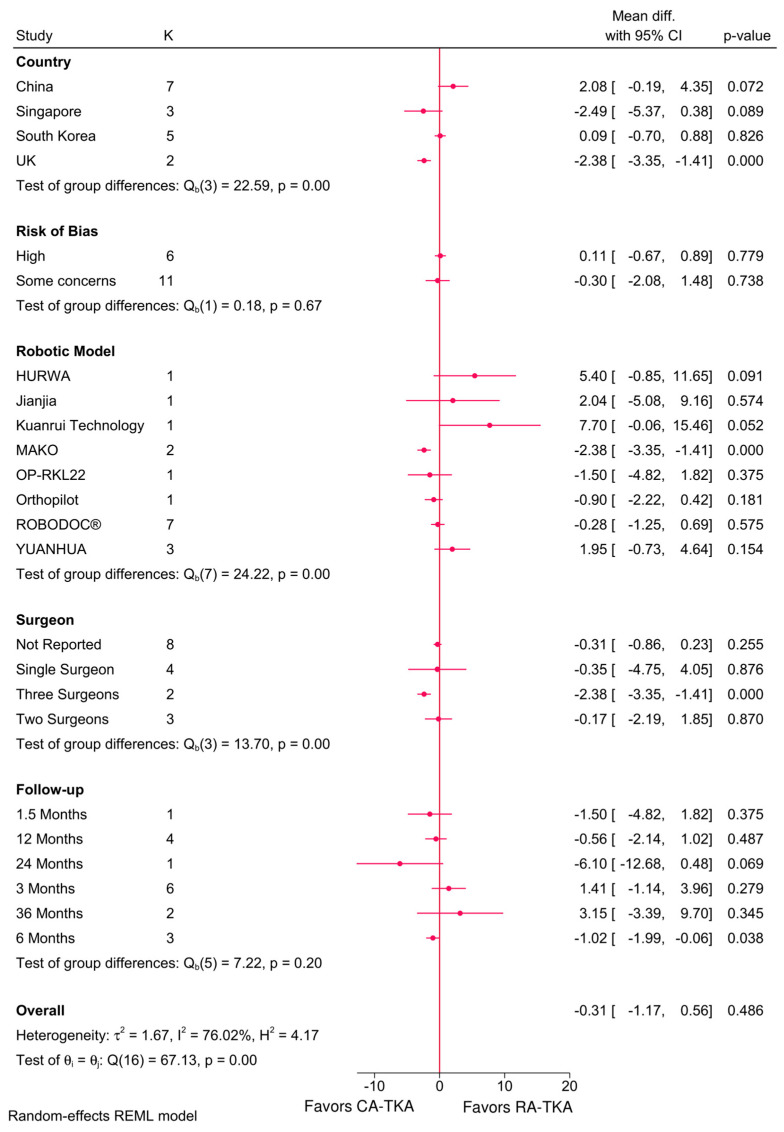
Subgroup analysis of the difference in KSS score between robot-assisted and conventional total knee arthroplasty based on country, risk of bias, robotic system, number of surgeons, and follow-up period.

**Figure 4:**
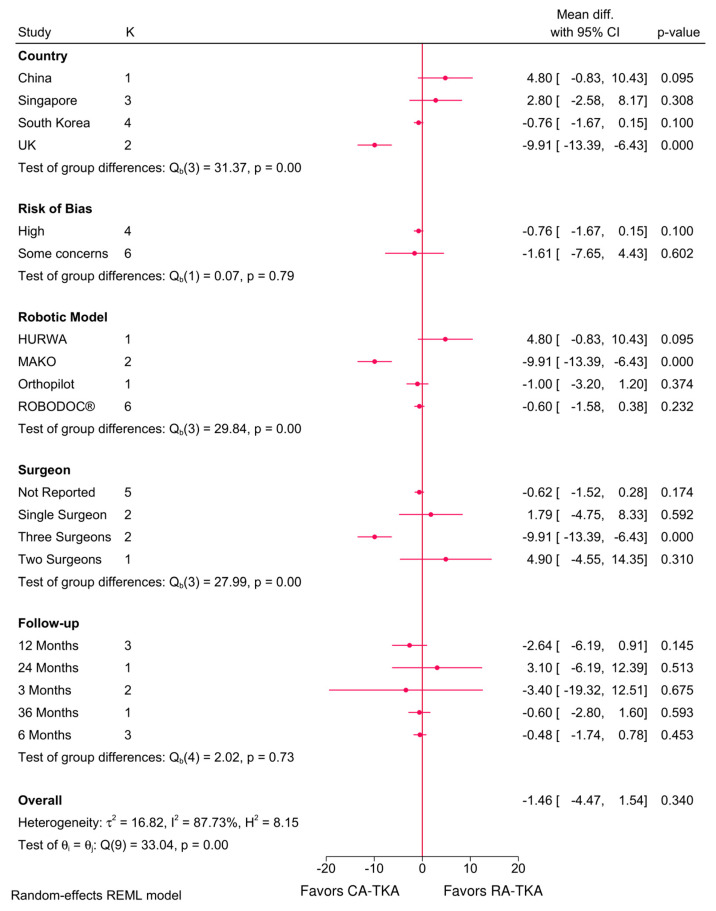
Subgroup analysis of the difference in KSS functional score between robot-assisted and conventional total knee arthroplasty based on country, risk of bias, robotic system, number of surgeons, and follow-up period.

The robotic system (*p* < 0.001), country (*p* < 0.001), and number of surgeons (*p* < 0.001) significantly influenced the KSS scores (Figure 3). RA-TKA resulted in significantly lower total KSS in the UK (MD = −2.38; 95%CI: −3.35, −1.41), with the MAKO system, when performed by three surgeons, and at six months (MD = −1.02; 95%CI: −1.99, −0.06). Functional KSS was also significantly lower in the UK (MD = −9.91; 95%CI: −13.39, −6.34), with the MAKO system and in three-surgeon cases (Figure 4). However, meta-regression identified no significant predictors for KSS scores (Table 2).

#### 3.4.2. OKS Score

None of the examined covariates significantly modified OKS scores (Figure S1), and no differences were observed between RA-TKA and C-TKA across country, risk of bias, robotic system, number of surgeons, or follow-up period. Meta-regression identified follow-up duration as the sole determinant of OKS (coefficient = 0.32, *p* = 0.009; Table 2).

#### 3.4.3. Pain Score

The number of surgeons was the only significant factor influencing postoperative pain (Figure S2). RA-TKA was associated with significantly lower pain scores when performed by two surgeons (MD = −1.10; 95%CI: −1.66, −0.54), with no other significant differences detected based on country, risk of bias, robotic system, or follow-up duration.

#### 3.4.4. ROM—Flexion and Extension

**Figure 5:**
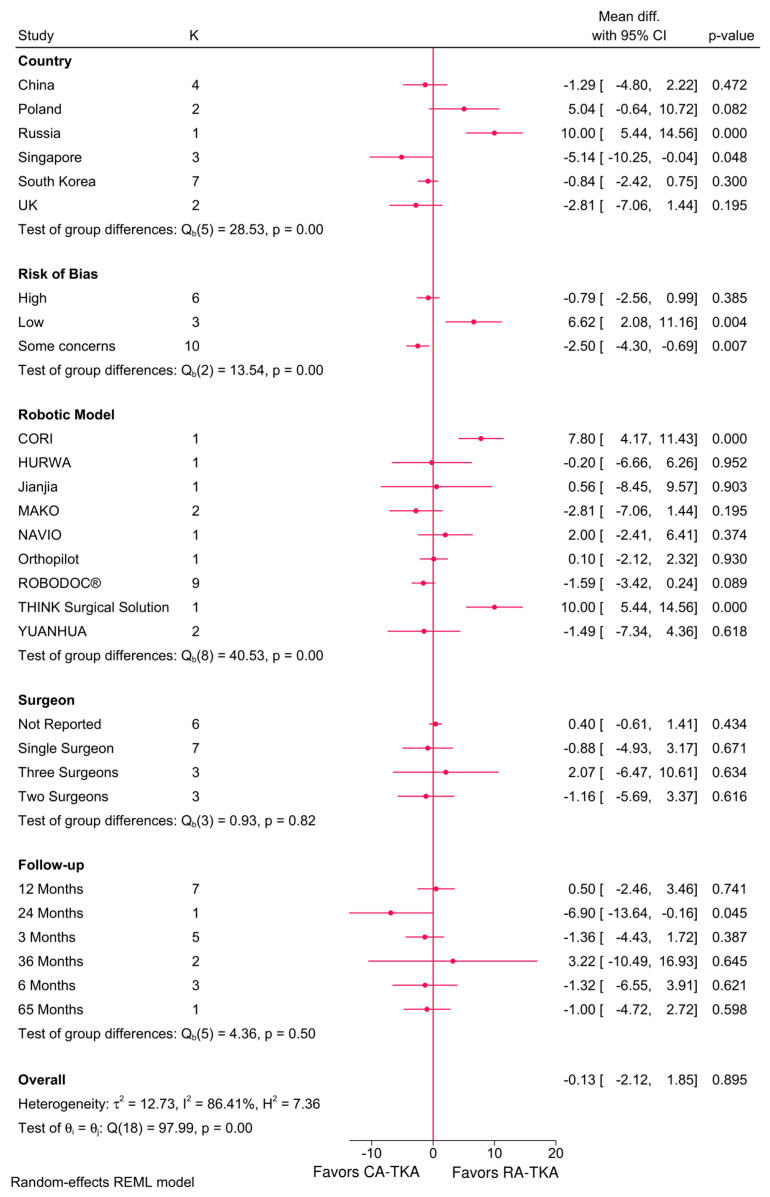
Subgroup analysis of the difference in ROM during flexion between robot-assisted and conventional total knee arthroplasty based on country, risk of bias, robotic system, number of surgeons, and follow-up period.

Significant effect modifiers of ROM in terms of flexion included robotic system (*p* = 0.00), country (*p* = 0.00), and risk of bias (*p* = 0.00; Figure 5). In Russia, RA-TKA achieved a 10.0° greater flexion than C-TKA, a difference that exceeds the commonly cited MCID for flexion (7–10°), suggesting potential clinical significance. In contrast, the −5.14° reduction observed in Singapore may reflect institutional differences in rehabilitation or system familiarity and falls below the threshold likely to impact function. Meta-regression indicated sample size (coefficient = −0.51, *p* = 0.047) and the NAVIO system (coefficient = −568.11, *p* = 0.049) as significant determinants (Table 2). No significant modifications were observed for ROM in terms of extension (Figure S3).

#### 3.4.5. HSS and WOMAC Scores

RA-TKA and C-TKA showed no significant differences in HSS (Figure S4) or WOMAC scores (Figure S5) across all examined subgroups. Meta-regression identified no significant predictors (Table 2).

#### 3.4.6. Operative Time

**Figure 6:**
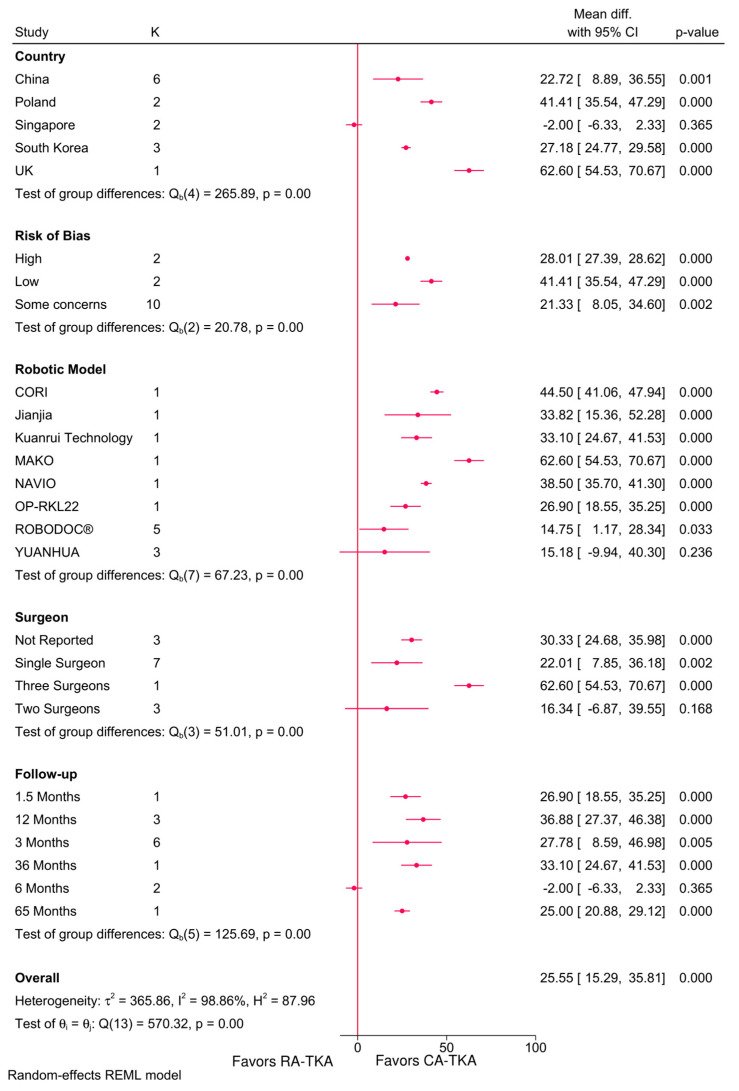
Subgroup analysis of the difference in operative time between robot-assisted and conventional total knee arthroplasty based on country, risk of bias, robotic system, number of surgeons, and follow-up period.

RA-TKA led to significantly longer operative times across all subgroups except for Singapore, YUANHUA robotic system (MD = 15.18; 95%CI: −9.94, 40.30), two-surgeon procedures, and at six months follow-up (Figure 6). Meta-regression confirmed that all robotic systems (NAVIO, MAKO, YUANHUA, and CORI) increased operative time compared to ROBODOC (*p* = 0.000), with the number of surgeons, risk of bias, and sample size also being significant determinants (Table 2).

#### 3.4.7. Intraoperative Blood Loss

RA-TKA and C-TKA had similar intraoperative blood loss, except in China, where RA-TKA was associated with significantly higher blood loss (MD = 21 mL; 95%CI: 2.11, 39.89), and in RCTs with some concerns (Figure S6). No significant predictors were found in the meta-regression analysis (Table 2).

#### 3.4.8. Satisfaction Rate

Satisfaction rates were generally similar between RA-TKA and C-TKA, except in South Korea (OR = 7.57; 95%CI: 1.60, 35.93), in high-risk RCTs (OR = 6.25; 95%CI: 1.33, 29.43), among two-surgeon cases, and at 65 months follow-up (OR = 7.58; 95%CI: 1.60, 35.93; Figure S7). Meta-regression identified the number of surgeons as a significant determinant (coefficient = 2.16, *p* = 0.018; Table 2).

#### 3.4.9. Complication Rate

No significant differences were observed in total complication rates between RA-TKA and C-TKA across all subgroups based on country (*p* = 0.73), risk of bias (*p* = 0.60), robotic system (*p* = 0.96), number of surgeons (*p* = 0.97), or follow-up period (*p* = 0.80; Figure S8).

**Figure 7:**
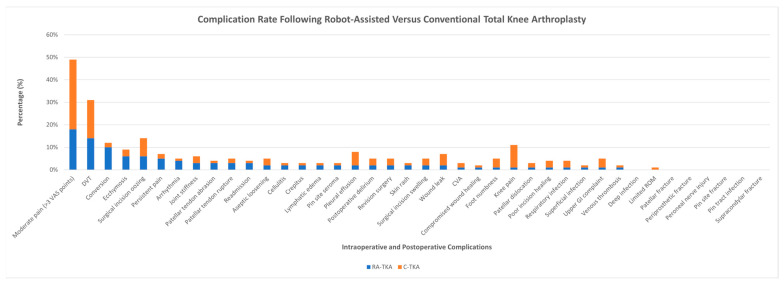
A graph showing the difference in the rates of all complications reported for robot-assisted and conventional total knee arthroplasty.

However, moderate pain (VAS > 3) was lower in RA-TKA (18%) compared to C-TKA (31%), and deep vein thrombosis (DVT) was slightly less frequent in RA-TKA (14% vs. 17%). Conversely, RA-TKA had a higher rate of conversion to open surgery (10% vs. 2%). Minor variations were noted for other complications (i.e., ecchymosis and surgical incision oozing). Persistent pain and arrhythmias were slightly more common in RA-TKA (Figure 7).

Joint stiffness rates were similar (3% in both groups), while patellar tendon complications, including abrasion (3% vs. 1%) and rupture (3% vs. 2%), were slightly more frequent in RA-TKA. Readmission rates were also higher in RA-TKA. Pleural effusion was more common in C-TKA (6% vs. 2%), as were wound leaks (5% vs. 2%), while surgical incision swelling remained comparable. Deep infections, patellar fractures, and supracondylar fractures were absent in both groups.

### 3.5. Radiographic Outcomes

**Figure 8:**
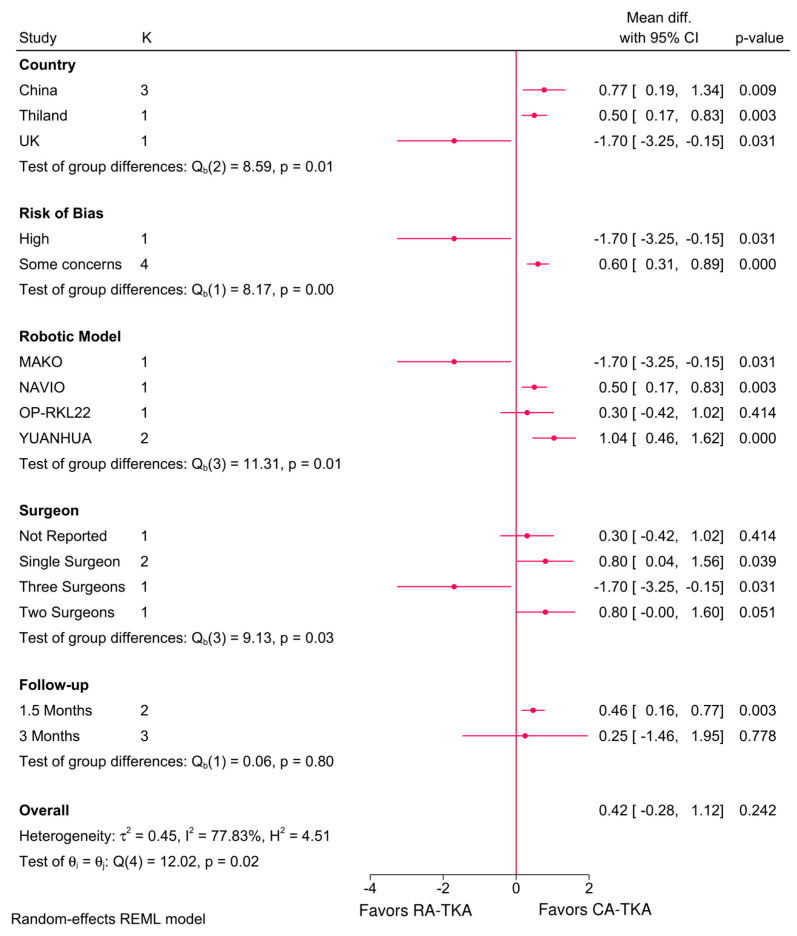
Subgroup analysis of the difference in HKA Angle between robot-assisted and conventional total knee arthroplasty based on country, risk of bias, robotic system, number of surgeons, and follow-up period.

The findings on radiographic outcomes are presented in Supplementary File S1 (Figure 8; Figures S9–S15). In brief, Figure 8 demonstrates that the HKA Angle was significantly influenced by robotic system, country, risk of bias, and number of surgeons. RA-TKA showed more favorable alignment in studies from China and Thailand and when using the NAVIO and YUANHUA systems, while alignment outcomes were less favorable in UK-based studies and with the MAKO system. These differences suggest system- and context-specific effects on implant positioning.

## 4. Discussion

This systematic review and meta-analysis provides a comprehensive comparison pf RA-TKA and C-TKA across clinical, radiographic, and safety outcomes. While robotic-assisted techniques have gained in popularity owing to their potential to improve implant positioning, the findings of this study highlight both the benefits and limitations of RA-TKA in clinical practice.

### 4.1. Clinical Outcomes

RA-TKA showed modest improvements in clinical outcomes but was not consistently superior. Notably, the MAKO system was associated with lower KSS scores in UK-based studies, which stands in contrast to its performance in other regions. This discrepancy may reflect variations in surgeon training, institutional learning curves, or early adoption phases. Since MAKO is a semi-active, CT-based system, its learning curve may be steeper compared to imageless platforms. These findings highlight the importance of real-world implementation factors when interpreting comparative device performance, and they underscore the need for future studies to stratify outcomes by surgeon experience and center volume [52,53].

OKS, a widely used patient-reported measure, showed no significant differences between RA-TKA and C-TKA, with follow-up duration as the only significant predictor. This suggests that while RA-TKA may not provide immediate benefits, it could offer long-term advantages as surgical proficiency improves. A reduction in postoperative pain, as reflected in VAS scores, was observed only in surgeries performed by two surgeons. This may reflect a moderate level of inter-operator variability, which could have inadvertently optimized consistency without the extremes seen in single-surgeon (potential overstandardization or learning effects) or three-surgeon (potential procedural inconsistency) cohorts. Alternatively, this could be a chance association, especially given the exploratory nature of our meta-regression. Regardless, it highlights the importance of considering operator heterogeneity when interpreting pooled outcomes and suggests an area for future focused research [54].

ROM outcomes were inconsistent, with RA-TKA linked to better flexion in Russia and low-risk trials but lower ROM in Singapore and studies with bias concerns. Sample size and the NAVIO system were significant determinants of flexion, highlighting the role of system-specific factors. The absence of significant differences in HSS scores further suggests that, despite theoretical advantages in precision and alignment, RA-TKA does not consistently yield superior functional outcomes in routine practice.

### 4.2. Radiographic Outcomes

Radiographic parameters, particularly the HKA Angle, provide objective measures of implant positioning and alignment, which are crucial for long-term prosthesis survival and patient satisfaction. This analysis found that RA-TKA achieved better alignment with the NAVIO and YUANHUA systems, while UK-based studies and those using MAKO reported inferior alignment compared to C-TKA. Meta-regression confirmed the MAKO system as a significant predictor of lower HKA Angles, suggesting system-specific limitations [55].

Other radiographic measures, such as FCIA and TCIA, varied by robotic system and study location. The MAKO system showed improved FCIA in certain settings, whereas TCIA was more favorable in the UK with MAKO. These results emphasize the role of robotic system selection in radiographic outcomes, with some systems providing more consistent alignment than others. FSIA and TSIA showed fewer differences, though NAVIO and CORI systems were associated with better TSIA outcomes. The variability in alignment suggests that multiple factors—including robotic system, surgeon experience, and patient selection—affect implant positioning precision.

While RA-TKA consistently demonstrated improved mechanical alignment (e.g., reduced HKA Deviation), the translation of this advantage into long-term clinical benefits remains complex. Optimal implant alignment is associated with reduced polyethylene wear and lower revision rates in registry data, particularly in younger and more active patients. However, our meta-analysis found no consistent improvements in PROMs, suggesting that radiographic precision alone may not predict short-term functional gains. These findings reinforce the need for longitudinal studies linking alignment accuracy to implant survival and patient-reported recovery over time

Notably, our subgroup analyses and meta-regression revealed inconsistent results across robotic platforms, highlighting that the clinical and radiographic benefits of RA-TKA are not uniform. For example, the NAVIO and YUANHUA systems were associated with significantly improved HKA alignment in specific regions, while the MAKO system, despite its widespread use, demonstrated less favorable results in some contexts—particularly in the UK subgroup. These differences may stem from variability in system mechanics (e.g., active vs. semi-active control), preoperative planning requirements (CT-based vs. imageless), and surgeon experience. Such findings underscore the need for system-specific trials and suggest that surgeon familiarity and context may be as important as the robotic platform itself.

### 4.3. Safety and Complications

The overall complication rate did not significantly differ between RA-TKA and C-TKA, supporting previous findings that robotic systems are generally safe in experienced surgical hands. However, RA-TKA was associated with higher rates of conversion to open surgery and persistent pain, possibly reflecting the learning curve or technical challenges specific to robotic-assisted procedures. Readmission rates were also higher in RA-TKA, suggesting that more frequent postoperative interventions may be required.

An important procedural limitation observed in some trials was the higher rate of intraoperative conversion to conventional techniques in the RA-TKA group. While not universally reported, these conversions likely stem from technical errors (e.g., image registration failure and robotic-arm malfunctions) or surgeon unfamiliarity with system workflows. Most conversions occurred in early-experience cohorts, emphasizing the importance of structured onboarding, case volume thresholds, and system-specific proficiency standards. These strategies may help reduce abandonment rates and preserve the benefits of robotic assistance.

Conversely, C-TKA was linked to higher rates of pleural effusion and wound leakage, indicating that RA-TKA may reduce certain complications due to improved surgical precision and reduced soft tissue trauma. These findings align with reports suggesting that robotic-assisted procedures can enhance surgical accuracy while minimizing unnecessary tissue disruption. However, further investigation is needed to determine the clinical significance of these differences and whether they translate into long-term benefits for patients [56].

### 4.4. Comparison with Other Systematic Reviews and Meta-Analyses

Table S3 summarizes the methodological and analytical scope of recent systematic reviews on robot-assisted versus conventional TKA. Compared to these prior studies, our review incorporates the largest patient cohort to date (5614 patients from 25 RCTs), utilizes advanced analytic methods including meta-regression and subgroup analysis, and evaluates a broader set of clinical, radiographic, and safety outcomes.

The findings of this study align with previous meta-analyses demonstrating that RA-TKA improves radiographic alignment while offering limited advantages in terms of clinical outcomes. Studies by Riangsomboon et al. [18] and Alrajeb et al. [19] reported similar enhancements in mechanical alignment and fewer outliers with RA-TKA, particularly in HKAs and Coronal Inclination Angles. These findings support the current analysis, which identified superior radiographic outcomes with RA-TKA in certain robotic systems. However, these studies, like the present review, found minimal differences in functional outcomes such as ROM, KSS, and OKS [18].

Similarly, Agarwal et al. [57] reported improved radiographic alignment with RA-TKA but no significant effects on pain or functional scores, reinforcing the notion that radiographic precision does not necessarily translate into better patient-reported outcomes. The study by Fozo et al. [15] noted a slight advantage in pain outcomes with RA-TKA, but the difference was not statistically significant. This aligns with the current findings, where pain reduction was observed only in specific subgroups, such as surgeries performed by two surgeons.

Regarding safety, Nogalo et al. [56] and Alshahrani [58] highlighted the potential complications associated with RA-TKA, such as iatrogenic injuries, pin-hole fractures, and higher readmission rates. In our study, we observed a higher conversion rate to open surgery in the RA-TKA group, aligning with the safety concerns raised in these reviews. However, other complications, such as joint stiffness and DVT, were comparable between RA-TKA and C-TKA, further supporting the overall safety of robotic systems when used by experienced surgeons.

### 4.5. Limitations and Future Directions

Several limitations should be noted. First, the variability in outcomes across different robotic systems and countries suggests that the generalizability of RA-TKA outcomes may be limited by the context in which the surgery is performed. Future studies should focus on head-to-head comparisons of different robotic systems to identify the most effective and reliable technologies. Second, risk of bias was often introduced through inadequate reporting of allocation concealment, lack of blinding (especially for outcome assessment), and incomplete follow-up. Third, the surgical expertise and number of operating surgeons were inconsistently reported, despite their clear influence on procedural outcomes—a factor we attempted to address through meta-regression. Fourth, heterogeneity in robotic platforms, implant types, and rehabilitation protocols complicated direct comparisons. Fifth, outcome measurement tools and reporting time points varied widely, particularly for patient-reported measures like OKS and WOMAC, limiting data harmonization. The relatively short follow-up durations in many trials hinder robust conclusions about long-term implant survivorship and functional recovery.

These methodological concerns suggest that while current evidence is promising, stronger and more standardized RCTs are needed. Identifying patient subgroups that are most likely to benefit from robotic assistance will be critical for optimizing outcomes. Finally, while the meta-regression analyses provided valuable insights into the predictors of outcomes, further exploration of surgeon-related factors, such as experience and training with robotic systems, is warranted.

## 5. Conclusions

Robotic-assisted TKA may offer radiographic alignment advantages and context-specific improvements in select patient-reported or surgical outcomes. However, these benefits are not uniformly observed across all platforms or settings and do not consistently extend to functional outcomes. Given the variability in system design, surgeon experience, and follow-up duration, caution is warranted in generalizing these findings. Successful implementation depends on adequate surgeon training and procedural volume. Future high-quality, system-specific RCTs are needed to clarify the long-term clinical impact of RA-TKA.

## Figures and Tables

**Table 1:** Baseline characteristics of RCTs comparing robotic to conventional total knee arthroplasty.

Author (YOP)	Study Design	Registration Number	Country	YOI	Sample	Sample		Robot Type	Age; M (SD)		Gender (M/F)		Surgeon Expertise	Cost Analysis	FI (Month)	
						RA-TKA	C-TKA		RA-TKA	C-TKA	RA-TKA	C-TKA			RA-TKA	C-TKA
Adamska (2022) [30]	RCT	NR	Poland	2022	215	76	68	NAVIO	66 (7.5)	65 (8.2)	22/42	30/35	An experienced arthroplasty surgeon	No	12	
					215	71		CORI	69 (6.8)		34/35					
Banger (2020) [31]	RCT	(ISRCTN 12151461)	UK	2020	70	38	32	MAKO	68.7 (7.8)	70.5 (7.1)	15/17	18/20	Three orthopedic surgeons with extensive experience in both TKA and robotic-assisted UKA	No	3	
Blyth (2021) [32]	RCT	(ISRCTN 12151461)	UK	2021	76	34	42	MAKO	68.7 (7.7)	70.4 (7.1)	17/17	21/21	Three surgeons with extensive experience in TKA, robotic-assisted, and computer-navigated knee surgery	No	12	
Cho (2019) [34]	RCT	NR	South Korea	2007	390	160	230	ROBODOC^®^	68.2 (3.83)	67.6 (4.17)	14/141	33/163	Not clarified	No	10.8 (0.9)	11.2 (1.1)
Kim (2020) [37]	RCT	NCT 03659318).	South Korea	2008	1348	674	674	ROBODOC^®^	60 (7)	61 (8)	132/542	144/530	The surgeon who performed the procedures in this report performed 30 robotic-assisted TKAs using the system	No	13 (0.83)	14 (0.83)
Lee (2023) [38]	RCT	NR	Korea	2009	855	194	270	ROBODOC^®^	71.8 (8.2)	71.0 (7)	18/176	20/250	Not clarified	No	11.9 (1.5)	11.8 (1.5)
					855	391		Orthopilot	71.6 (8.1)	71.0 (7)	26/365				12 (1.4)	11.8 (1.5)
Li (2022) [39]	RCT	NR	China	2020	150	73	77	HURWA	68.0 (7.97)	69.0 (6.00)	13/60	15/62	All operations were performed by experienced surgeons	No	3	
Liow (2017) [40]	RCT	NR	Singapore	2012	60	31	29	ROBODOC^®^	-	-	-	-	A single experienced surgeon	No	24	
Liu (2024) [42]	RCT	ChiCTR2200065786)	China	2021	88	44	44	-	71.75 (2.067)	72.25 (2.067)	11/0	12/0	Not clarified	No	3	
Lychagin (2024) [43]	RCT	NR	Russia	2019	118	56	62	THINK Surgical Solution	67.7 (10.2)	66.5 (8.7)	12/44	15/47	Three experienced surgeons performed the surgical procedures in all groups	No	36	
Park (2007) [44]	RCT	NR	South Korea	2007	62	32	30	ROBODOC^®^	62.7 (6.51)	67.8 (6.44)	-	-	Not clarified	No	45.0 (0.69)	49.3 (3.47)
Song (2011) [45]	RCT	NR	Korea	2004	30	15	15	ROBODOC^®^	-	-	0/15	0/15	Single surgeon who had experience of more than 150 cases of robot-assisted TKA	Yes	16 (3.2)	
Song (2013) [46]	RCT	NR	Korea	2004	100	50	50	ROBODOC^®^	66.1 (7.1)	64.8 (5.3)	4/46	5/45	One surgeon experienced in both conventional TKA techniques and the ROBODOC system for TKA	No	65 (4.25)	65 (10)
Thiengwittayaporn (2021) [47]	RCT	NCT04307251	Thiland	2020	152	75	77	NAVIO	69.0 (8.3)	69.1 (7.3)	6/69	15/62	One experienced surgeon who had experience in computer navigation and the conventional jig-based instruments	No	1.5	
Xu (2022) [51]	RCT	(ChiCTR2000031282)	China	2022	32	16	16	YUANHUA	66.6 (3.7)	67.3 (3.5)	3/14	3/13	All the surgeries were completed by the same senior surgeon	No	3	
Xu (2022) [50]	RCT	ChiCTR2100042323)	China	2020	72	37	35	YUANHUA	64.5 (5.3)	63.4 (7.2)	11/26	7/28	Two senior joint surgeons	No	3	
Vaidya (2020) [49]	RCT	NR	India	2020	60	32	28	NAVIO	62.2 (10)	59.9 (8)	8/24	4/24	Not clarified	No	3	
Tian (2023) [48]	RCT	ChiCTR2200065786	China	2021	123	62	61	Jianjia	68.17 (7.59)	68.84 (7.12)	13/49	15/46	Not clarified	No	3	
Bollars (2023) [33]	RCT	NR	Belgium	2021	52	26	26	NAVIO	64.4 (8.7)	66.4 (7.2)	11/15	9/17	Not clarified	No	1.5	
Clement (2023) [35]	RCT	(ISRCTN 47889316)	UK	2020	87	46	41	MAKO	66.8 (8.7)	66.7 (9.6)	26/24	21/29	Two surgeons, both of whom had over 20 years’ experience as primary and revision knee arthroplasty surgeons	Yes	6	
Li (2023) [24]	RCT	NR	China	2023	134	68	66	Kuanrui Technology	64.5 (6.3)	65.0 (5.5)	9/57	16/45	Not clarified	No	36	
Geng (2024) [36]	RCT	NR	China	2023	130	65	65	OP-RKL22	68.1 (5)	67.4 (5.5)	16/49	21/44	Not clarified	No	1.5	
Liow (2014) [41]	RCT	NR	Singapore	2012	60	31	29	ROBODOC^®^	67.5 (8.6)	68.3 (7.7)	-	-	Two surgeons performed all operations	No	6	
Airapetov (2023) [23]	RCT	NR	Russia	2023	20	10	10	-	61.4 (14.7)	63.4 (14.7)	4/6	3/7	Single surgeon performed all operations	No	0.33	
Yuan (2021) [25]	RCT	NR	China	2020	60	28	32	YUANHUA	65.2	65.4	9/19	4/28	Two surgeons performed all operations	No	3	

YOP: year of publication; NR: not reported; YOI: year of investigation; RA-TKA: robot-assisted total knee arthroplasty; C-TKA: conventional total knee arthroplasty; M: mean; SD: standard deviation; M/F: male/female; UK: United Kingdom.

**Table 2:** Meta-regression analysis showing the determinants of clinical and radiographic outcomes in patients undergoing RA-TKA versus C-TKA.

	KSS		OKS		ROM—Flexion		ROM—Extension		HSS		WOMAC	
	Coefficient	*p*-Value	Coefficient	*p*-Value	Coefficient	*p*-Value	Coefficient	*p*-Value	Coefficient	*p*-Value	Coefficient	*p*-Value
**Robotic System [Reference Group: ROBODOC]**												
NAVIO	-	-	-	-	−568.11	0.049	−0.30	0.585	-	-	-	-
MAKO	−279.33	0.402	-	-	−31.23	0.305	-	-	-	-	−475.99	0.475
YUANHUA	−282.42	0.413	-	-	−36.42	0.285	-	-	−1.40	0.950	−39.67	0.701
CORI	-	-	-	-	−562.31	0.051	-	-	-	-	-	-
THINK Surgical Solution	-	-	-	-	−601.71	0.055	-	-	-	-	-	-
**Risk of Bias [Reference Group: Low Risk]**												
Some concerns	-	-	-	-	−612.79	0.055	-	-	-	-	−452.60	0.486
**Number of Surgeons [Reference Group: Single Surgeon]**												
Two surgeons	4.90	0.644	1.73	0.275	-	-	-	-	-	-	30.03	0.072
**Follow-up (month)**	−0.07	0.506	0.32	0.009	−0.31	0.561	-	-	-	-	0.10	0.949
**Sample size (per patient)**	−0.22	0.408	-	-	−0.51	0.047	-	-	-	-	−0.36	0.487
**Constant**	293.95	0.405	−2.31	0.031	682.60	0.047	0.30	0.244	1.00	0.941	480.83	0.489
	**Operative Time**		**Intraoperative Blood Loss**		**Satisfaction Rate**		**Complications**		**HKA Angle**		**HKA Deviation**	
	**Coefficient**	***p*** **-Value**	**Coefficient**	***p*** **-Value**	**Coefficient**	***p*** **-Value**	**Coefficient**	***p*** **-Value**	**Coefficient**	***p*** **-Value**	**Coefficient**	***p*** **-Value**
**Robotic System [Reference Group: ROBODOC]**												
NAVIO	2446.45	<0.0001	-	-	-	-	0.59	0.780	−0.80	0.084	-	-
MAKO	89.20	<0.0001	-	-	2.85	0.566	−2.44	0.237	−3.00	0.001	-	-
YUANHUA	115.70	0.004	4691.57	0.693	-	-	−0.21	0.869	-	-	0.97	0.009
CORI	2452.45	<0.0001	-	-	-	-	0.65	0.756	-	-	-	-
THINK Surgical Solution	-	-	-	-	-	-	−3.31	0.275	-	-	-	-
**Number of Surgeons [Reference Group: Single Surgeon]**												
Two surgeons	−41.40	0.030	−55.63	0.830	2.16	0.018	0.36	0.678	−0.50	0.400	-	-
Three surgeons	-	-	-	-	-	-	2.73	0.134	-	-	-	-
**Risk of Bias [Reference Group: Low Risk]**												
Some concerns	2680.20	<0.0001	-	-	-	-	-	-	-	-	-	-
**Follow-up (month)**	-	-	-	-	0.09	0.311	−0.04	0.693	-	-	-	-
**Sample size (per patient)**	2.15	<0.0001	3.58	0.691	-	-	-	-	-	-	-	-
**Constant**	−2870.20	<0.0001	−4824.33	0.692	−3.51	0.525	−0.25	0.843	1.30	0.003	−1.87	<0.0001
	**FCIA**		**TCIA**		**FSIA**		**TSIA**		**TTA**			
	**Coefficient**	***p*** **-Value**	**Coefficient**	***p*** **-Value**	**Coefficient**	***p*** **-Value**	**Coefficient**	***p*** **-Value**	**Coefficient**	***p*-Value**		
**Robotic System [Reference Group: ROBODOC]**												
NAVIO	1.50	0.492	3.37	0.080	−3.93	0.312	−1.67	<0.0001	-	-		
MAKO	−63.40	0.232	−57.25	0.218	10.55	0.912	3.80	<0.0001	−6.90	<0.0001		
YUANHUA	−6.00	0.041	−1.03	0.683	−0.27	0.918	-	-	-	-		
CORI	-	-	-	-	-	-	−1.82	<0.0001	-	-		
**Number of Surgeons [Reference Group: Single Surgeon]**												
Two surgeons	2.70	0.082	0.37	0.768	-	-	-	-	-	-		
**Risk of Bias [Reference Group: Low Risk]**												
Some concerns	−61.90	0.232	−53.60	0.238	9.40	0.919	-	-	-	-		
**Sample size (per patient)**	−0.05	0.229	−0.04	0.252	0.01	0.911	-	-	-	-		
**Constant**	68.40	0.222	57.17	0.244	−10.23	0.919	0.001	1.000	0.001	1.000		

ROM: range of motion; KSS: Knee Society Score; OKS: Oxford Knee Score; HSS: Hospital for Special Surgery; HKA: Hip–Knee–Ankle; WOMAC: Western Ontario and McMaster Universities Arthritis Index; FCIA: Femoral Coronal Inclination Angle; TCIA: Tibial Coronal Inclination Angle; FSIA: Femoral Sagittal Inclination Angle; TSIA: Tibial Sagittal Inclination Angle; TTA: Transverse Tibial Angle.

## Data Availability

The original contributions presented in this study are included in the article/Supplementary Materials. Further inquiries can be directed to the corresponding author.

## Supplementary Materials

The following supporting information can be downloaded at: https://www.mdpi.com/article/10.3390/jcm14093249/s1.

## Abbreviations

AMSTAR	Assessing the Methodological Quality of Systematic Reviews
C-TKA	Conventional Total Knee Arthroplasty
CI	Confidence Interval
FCIA	Femoral Coronal Inclination Angle
FSIA	Femoral Sagittal Inclination Angle
HKA	Hip–Knee–Ankle
HSS	Hospital for Special Surgery
KSS	Knee Society Score
MD	Mean Difference
NAVIO	Name of a robotic system used in TKA
OKS	Oxford Knee Score
OR	Odds Ratio
PRISMA	Preferred Reporting Items for Systematic Reviews and Meta-Analyses
RA-TKA	Robot-Assisted Total Knee Arthroplasty
RCT	Randomized Controlled Trial
REM	Restricted Maximum Likelihood
ROB-2	Revised Cochrane Risk of Bias Tool
ROM	Range of Motion
TCIA	Tibial Coronal Inclination Angle
TSIA	Tibial Sagittal Inclination Angle
TFA	Transverse Femoral Angle
TKA	Total Knee Arthroplasty
VAS	Visual Analogue Scale
YUANHUA	Name of a robotic system used in TKA
