# Supplementary material for: Comparative Efficacy and Precision of Robot-Assisted vs. Conventional Total Knee Arthroplasty: A Systematic Review and Meta-Analysis of Randomized Controlled Trials
Source: J Clin Med. 2025 May 7;14(9):3249. doi: 10.3390/jcm14093249 (PMC12072899; doi:10.3390/jcm14093249)
Supplement: Supplementary file 1 [file jcm-14-03249-s001.zip › Supplemental Digital Content.pdf]

Comparative Efficacy and Precision of Robot-Assisted vs. Conventional Total Knee Arthroplasty: A Systematic Review and Meta-Analysis of Randomized Controlled Trials

Table S1. The detailed search criteria employed in our literature search

Database	No	Search Query	Results
**PubMed**	#1	Robot[tiab] OR Robotic[tiab] OR Robot-Assisted[tiab] OR "Robotic Surgical Procedures"[Mesh]	72,454
	#2	"Total Knee Arthroplasty"[tiab] OR TKA[tiab] OR "Arthroplasty, Replacement, Knee"[Mesh]	43,621
	#3	Random*[tiab] OR "Randomized Controlled Trial" [Publication Type]	1,692,646
	#4	#1 AND #2 AND #3	126
**Scopus**	#1	TITLE-ABS-KEY (Robot) OR TITLE-ABS-KEY (Robotic) OR TITLE-ABS-KEY (Robot-Assisted)	707,238
	#2	TITLE-ABS-KEY ("Total Knee Arthroplasty") OR TITLE-ABS-KEY (TKA)	39,674
	#3	TITLE-ABS-KEY (Random*)	3,416,158
	#4	#1 AND #2 AND #3	150
**Web of Science**	#1	AB=Robot OR AB=Robotic OR AB=Robot-Assisted	352,218
	#2	AB="Total Knee Arthroplasty" OR AB=TKA	24,081
	#3	AB=Random*	2,263,011
	#4	#1 AND #2 AND #3	90
**CENTRAL**	#1	Robot OR Robotic OR Robot-Assisted	7,993
	#2	"Total Knee Arthroplasty" OR TKA	8,029
	#3	Random*	1,468,040
	#4	#1 AND #2 AND #3	143
**Clinicaltrials.gov**	Condition/disease	Total Knee Arthroplasty	-
	Other terms	-	-
	Intervention/treatment	Robotic Surgery	-
	Final results	Completed Trials	13

**Google Scholar**	With all of the words	robotic random	-
	With the exact phrase	total knee arthroplasty	-
	With at least one of the words	-	-
	Total	Only the first 200 records were selected	200

Table S2. A list of excluded articles during the full-text screening phase

ID	Author	YOP	Title	Decision
**1**		2023	Functionally Aligned Total Knee Arthroplasty Restores Native Medial Pivot More Frequently Than Mechanically Aligned Total Knee Arthroplasty – A Prospective Randomized Trial	Conference Abstract
**49**	N. D. G. Clement, S. Baron, J. Smith, K. Weir, D. J. Deehan, D. J.	2024	Patients undergoing robotic arm- assisted total knee arthroplasty have a greater improvement in knee- specific pain but not in function A 12-MONTH REPORT FROM THE ROAM RANDOMIZED CONTROLLED TRIAL	Conference Abstract
**61**	L. D. Dorr	2020	CORR Insights®: does Robotic-assisted TKA Result in Better Outcome Scores or Long-Term Survivorship Than Conventional TKA? A Randomized, Controlled Trial	Duplicate
**73**	X. Z. Geng, Y. H. Li, Y. Zhao, M. W. Liu, Y. Q. Li, Z. J. Cai, H. Zhang, M. Yan, X. F. Sun, Z. W. Lv, X. Guo, F. Li, F. Tian, H.	2024	Early Radiographic and Clinical Outcomes of Robotic-arm-assisted <i>versus</i> Conventional Total Knee Arthroplasty: A Multicenter Randomized Controlled Trial	Duplicate
**119**	Z. C. Li, X. Wang, X. Zhang, B. Wang, W. Fan, Y. Yan, J. Zhang, X. Zhao, Y. Lin, Y. et al.,	2022	HURWA robotic-assisted total knee arthroplasty improves component positioning and alignment - A prospective randomized and multicenter study	Duplicate
**236**	S. W. Z. Young, N. Tay, M. L. Fulker, D. Esposito, C. Carter, M. Bayan, A. Farrington, B. Van Rooyen, R. Walker, M.	2022	A prospective randomised controlled trial of mechanical axis with soft tissue release balancing vs functional alignment with bony resection balancing in total knee replacement—a study using Stryker Mako robotic arm-assisted technology	Duplicate
**94**	B. K. Kayani, S. Tahmassebi, J. Oussedik, S. Moriarty, P. D. Haddad, F. S.	2020	A prospective double-blinded randomised control trial comparing robotic arm-assisted functionally aligned total knee arthroplasty versus robotic arm-assisted mechanically aligned total knee arthroplasty	Irrelevant comparison (functional vs mechanically aligned TKA)

**224**	D. Y. Wang, Y. Liang, X. Li, K. Huang, W.	2024	Enhancing total knee arthroplasty outcomes: the role of individualized femoral sagittal alignment in robotic-assisted surgery - A randomized controlled trial	Irrelevant comparison (individualized vs. default alignment)
**235**	S. W. Z. Young, N. Tay, M. L. Fulker, D. Esposito, C. Carter, M. Bayan, A. Farrington, B. Van Rooyen, R. Walker, M.	2022	A prospective randomised controlled trial of mechanical axis with soft tissue release balancing vs functional alignment with bony resection balancing in total knee replacement-a study using Stryker Mako robotic arm-assisted technology	Irrelevant comparison (mechnical vs. functional alignment)
**9**	B. R. Ajekigbe, J. Clement, N. Galloway, S. Gabrov, N. Smith, K. Weir, D. Deehan, D.	2024	Robotic-arm assisted versus manual total knee arthroplasty: functional gait analysis from a randomised controlled trial	Irrelevant outcome (Gait and sway analysis)
**66**	A. K. Fontalis, B. Asokan, A. Haddad, I. C. Tahmassebi, J. Konan, S. Oussedik, S. Haddad, F. S.	2022	Inflammatory Response in Robotic-Arm-Assisted Versus Conventional Jig-Based TKA and the Correlation with Early Functional Outcomes: results of a Prospective Randomized Controlled Trial	Irrelevant outcome (inflammatory markers)
**195**	A. B. Saad, S. Kayani, B. Plastow, R. Ollivier, M. Davis, E. Sharma, A.	2024	Robotic arthroplasty software training improves understanding of total knee arthroplasty alignment and balancing principles: a randomized controlled trial	RA-TKA training assessment
**391**	Winnock de Grave	2023	Clinical outcomes in TKA are enhanced by both robotic assistance and patient specific alignment: a comparative trial in 120 patients	Retrospective cohort
**3**	Actrn	2016	MAKO RAPTOR study-Efficacy of robot assisted partial knee replacement versus navigated total knee replacement	Study protocol
**4**	Actrn	2020	A prospective, randomized, controlled trial of Mechanical Axis with Soft Tissue Release Balancing vs Functional Alignment with Bony Release Balancing in Total Knee Replacement â€“ A study using Stryker Mako Robotic-Arm Assisted TechnologyÂ®	Study protocol
**36**	ChiCtr	2021	Robot-assisted control study of early clinical results of functional alignment and mechanical axis alignment for total knee arthroplasty: a prospective double-blind randomized controlled trial	Study protocol

**168**	Nct	2024	A Randomized Controlled Trial of the Sagittal Alignment Difference Between Mako Robotic TKA and Manual TKA	Study protocol
**179**	O. Nl	2023	Migration in a Cruciate Retaining and a Condylar Stabilizing Insert of a robot-assisted Uncemented Total Knee Prosthesis using Model-based RSA: a Mono-Center Randomized Controlled Trial with 10 years follow-up	Study protocol
**112**	Y. H. X. Lai, H. Su, Q. Wan, X. F. Yuan, M. C. Zhou, Z. K.	2022	Effect of tourniquet use on blood loss, pain, functional recovery, and complications in robot-assisted total knee arthroplasty: a prospective, double-blinded, randomized controlled trial	Torniquet in robotic
**14**	M. S. D. Banger, J. Jones, B. G. MacLean, A. D. Rowe, P. J. Blyth, M. J. G.	2022	Are there functional biomechanical differences in robotic arm-assisted bi-unicompartmental knee arthroplasty compared with conventional total knee arthroplasty? A prospective, randomized controlled trial	Unicompartmental knee arthroplasty
**13**	M. D. Banger, J. Rowe, P. Jones, B. MacLean, A. Blyth, M. J. B.	2021	Robotic arm-assisted versus conventional medial unicompartmental knee arthroplasty: five-year clinical outcomes of a randomized controlled trial	Unicompartmental knee arthroplasty
**18**	C. L. Batailler, T. Naaim, A. Servien, E. Cheze, L. Lustig, S.	2023	No difference of gait parameters in patients with image-free robotic-assisted medial unicompartmental knee arthroplasty compared to a conventional technique: early results of a randomized controlled trial	Unicompartmental knee arthroplasty
**19**	S. W. A. Bell, I. Jones, B. MacLean, A. Rowe, P. Blyth, M.	2016	Improved Accuracy of Component Positioning with Robotic-Assisted Unicompartmental Knee Arthroplasty: data from a Prospective, Randomized Controlled Study	Unicompartmental knee arthroplasty
**20**	S. W. A. Bell, I. Jones, B. MacLean, A. Rowe, P. Blyth, M.	2016	Improved Accuracy of Component Positioning with Robotic-Assisted Unicompartmental Knee Arthroplasty	Unicompartmental knee arthroplasty
**51**	J. H. Cobb, J. Gomes, P. Harris, S. Jakopec, M. Rodriguez, F. Barrett, A. Davies, B.	2006	Hands-on robotic unicompartmental knee replacement: a prospective, randomised controlled study of the acrobot system	Unicompartmental knee arthroplasty

**74**	A. M. Gilmour, A. D. Rowe, P. J. Banger, M. S. Donnelly, I. Jones, B. G. Blyth, M. J. G.	2018	Robotic-Arm-Assisted vs Conventional Unicompartmental Knee Arthroplasty. The 2-Year Clinical Outcomes of a Randomized Controlled Trial	Unicompartmental knee arthroplasty
**75**	A. M. Gilmour, A. D. Rowe, P. J. Banger, M. S. Donnelly, I. Jones, B. G. Blyth, M. J. G.	2018	Robotic-Arm–Assisted vs Conventional Unicompartmental Knee Arthroplasty. The 2-Year Clinical Outcomes of a Randomized Controlled Trial	Unicompartmental knee arthroplasty
**95**	B. K. Kayani, S. Tahmassebi, J. Ayuob, A. Moriarty, P. D. Haddad, F. S.	2020	Robotic-arm assisted medial unicondylar knee arthroplasty versus jig-based unicompartmental knee arthroplasty with navigation control: study protocol for a prospective randomised controlled trial	Unicompartmental knee arthroplasty

Table S3. A summary of the prior published systematic reviews and meta-analyses comparing R-TKA to C-TKA

Review (YOP)	Studies	Patients	Outcomes	Studies included in analysis	Results (R-TKA vs. C-TKA)
**RUANGSOMBOON (2023)**	12 RCTs	2200	WOMAC Score	5	NS
			KSS Score	5	NS
			HSS Score	3	NS
			ROM	8	NS
			Implant survivorship	1	NS
			HKA deviation	6	Sign. Reduction
			Deviation >3 degree	8	Sign. Reduction
			Intraoperative blood loss	4	NS
			Operative time	7	Sign. Increase
			LOS	2	NS
**ALSHAHRANI (2024)**	12 RCTs/NRSI	N/A	HSS Score	4	NS
			KSS Score	4	NS
			WOMAC Score	6	NS
			VAS score	2	NS
			Intraoperative blood loss	3	NS
			Readmission rate	2	NS
**Fozo (2023)**	9 RCTs/17 NRSI	N/A	HKA deviation	10	Sign. Increase
			HSS Score	7	NS
			OKS score	3	NS
			ROM	12	NS
			Operative time	9	Sign. Increase
			Tourniquet time	3	Sign. Increase
			WOMAC Score	7	NS
**Alrajeb (2024)**	7 RCTs	1942 knees	KSS Score	2	NS
			HSS Score	2	NS
			WOMAC Score	2	NS
			ROM	4	NS
			Complications	3	NS

			Tibiofemoral axis	3	NS
			Lateral tibial angle	6	NS
**Agarwal (2020)**	6 RCTs / 16 NRSI	2346	HSS Score	4	Sign. Reduction
			KSS Score	5	NS
			ROM	8	NS
			WOMAC Score	6	Sign. Increase
			Deviation >3 degree	7	Sign. Increase

YOP: year of publication; RCT: randomized controlled trial; NRSI: non-randomized study of intervention; NS: not significant; N/A: not available; ROM: range of motion; KSS: knee society score; HSS: hospital for special surgery; WOMAC: western Ontario and McMaster universities osteoarthritis index; HKA: hop-knee-ankle angle; LOS: length of hospital stay; OKS: oxford knee score.

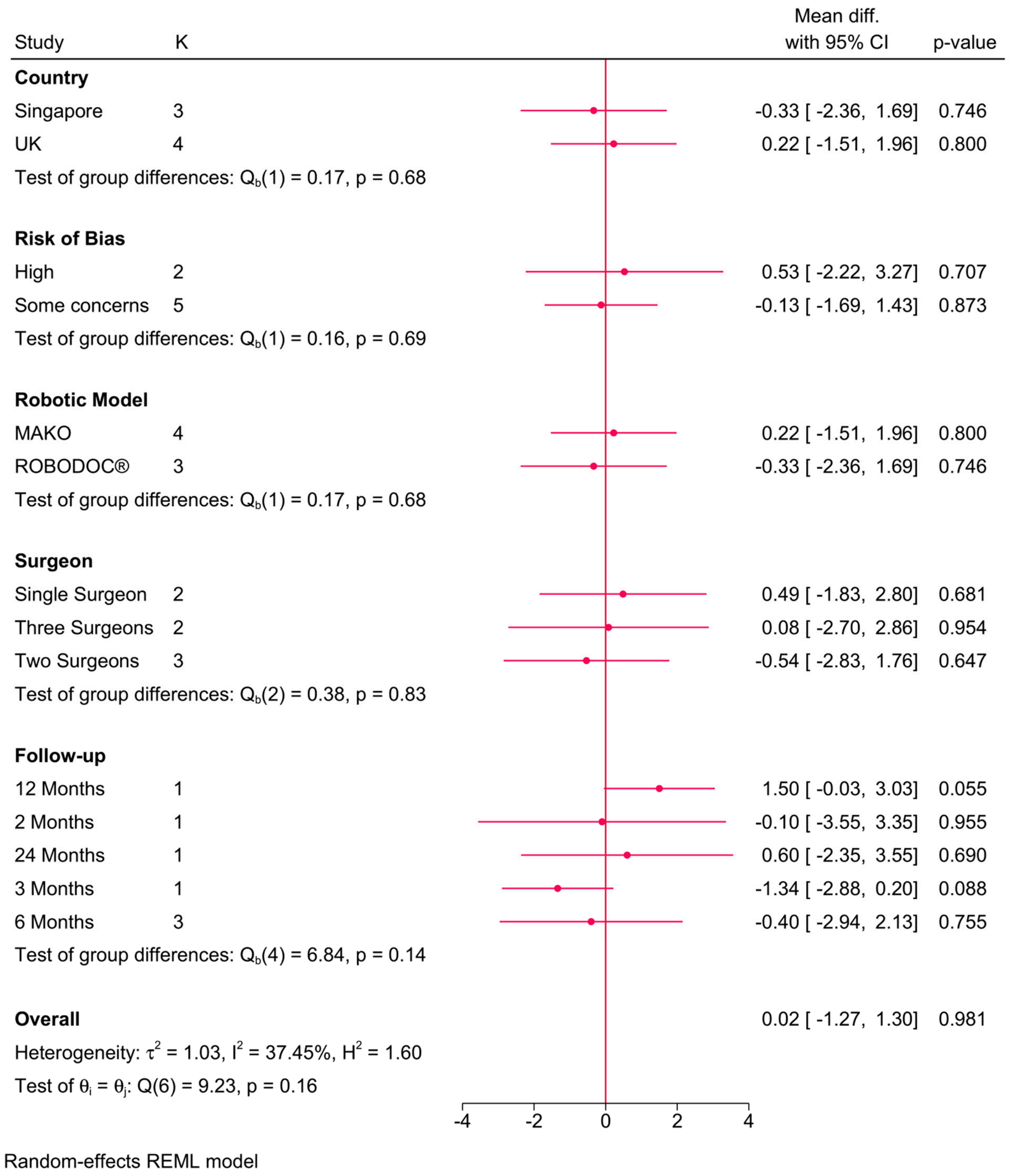

Figure S1. Subgroup analysis of the difference in OKS score between robot-assisted and conventional total knee arthroplasty based on country, risk of bias, robotic system, number of surgeons, and follow-up period

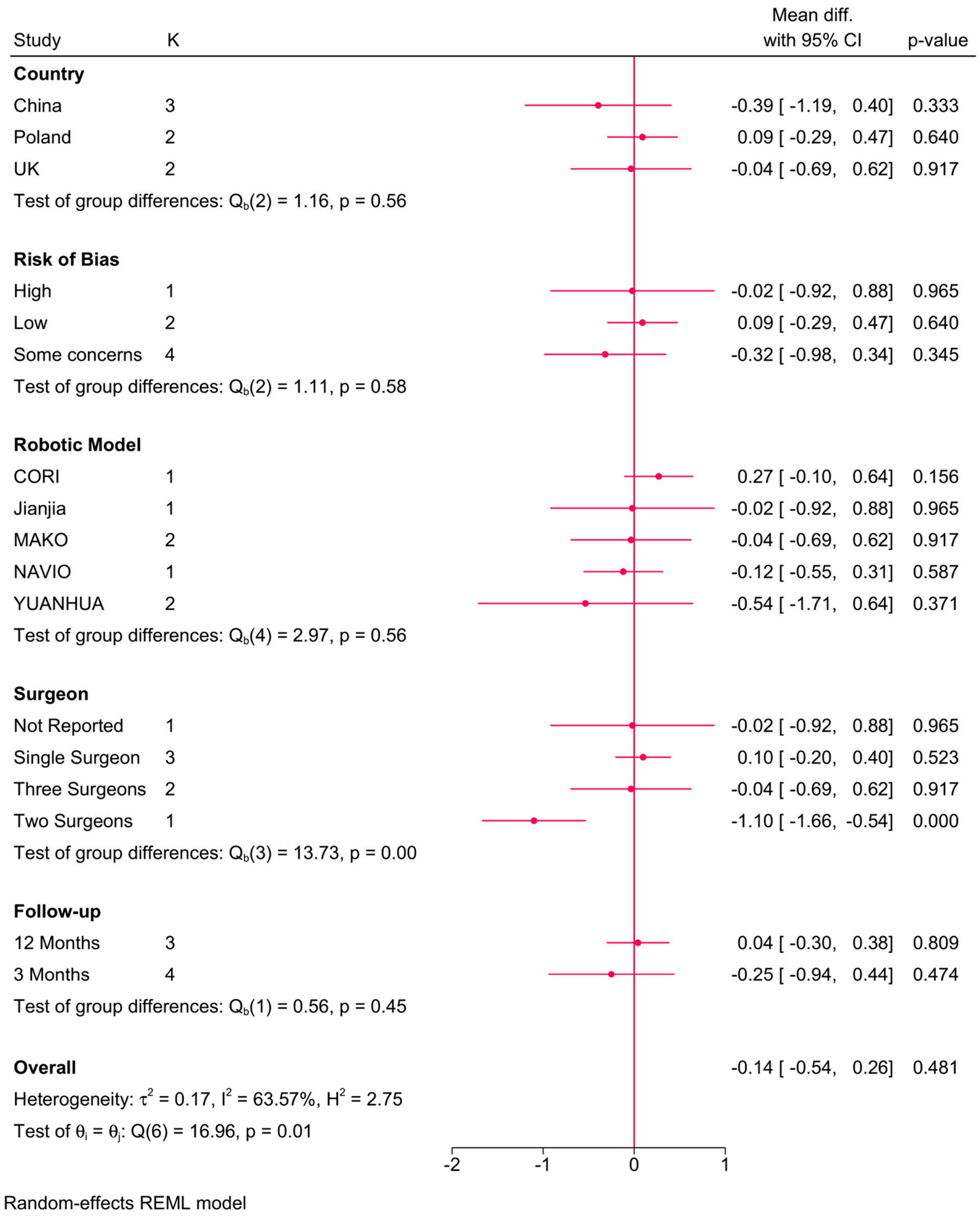

Figure S2. Subgroup analysis of the difference in pain (VAS) score between robot-assisted and conventional total knee arthroplasty based on country, risk of bias, robotic system, number of surgeons, and follow-up period

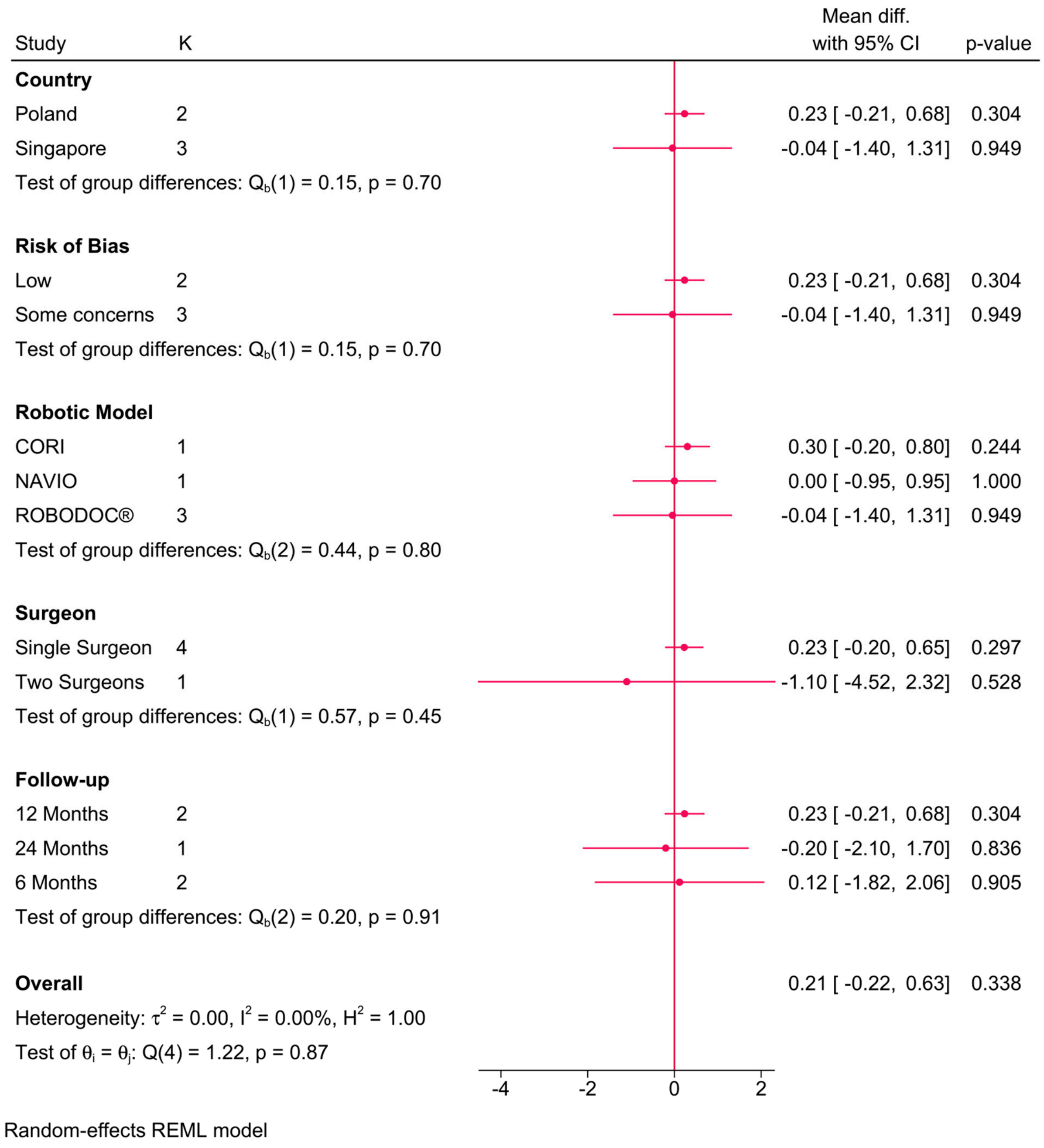

Figure S3. Subgroup analysis of the difference in ROM during extension between robot-assisted and conventional total knee arthroplasty based on country, risk of bias, robotic system, number of surgeons, and follow-up period

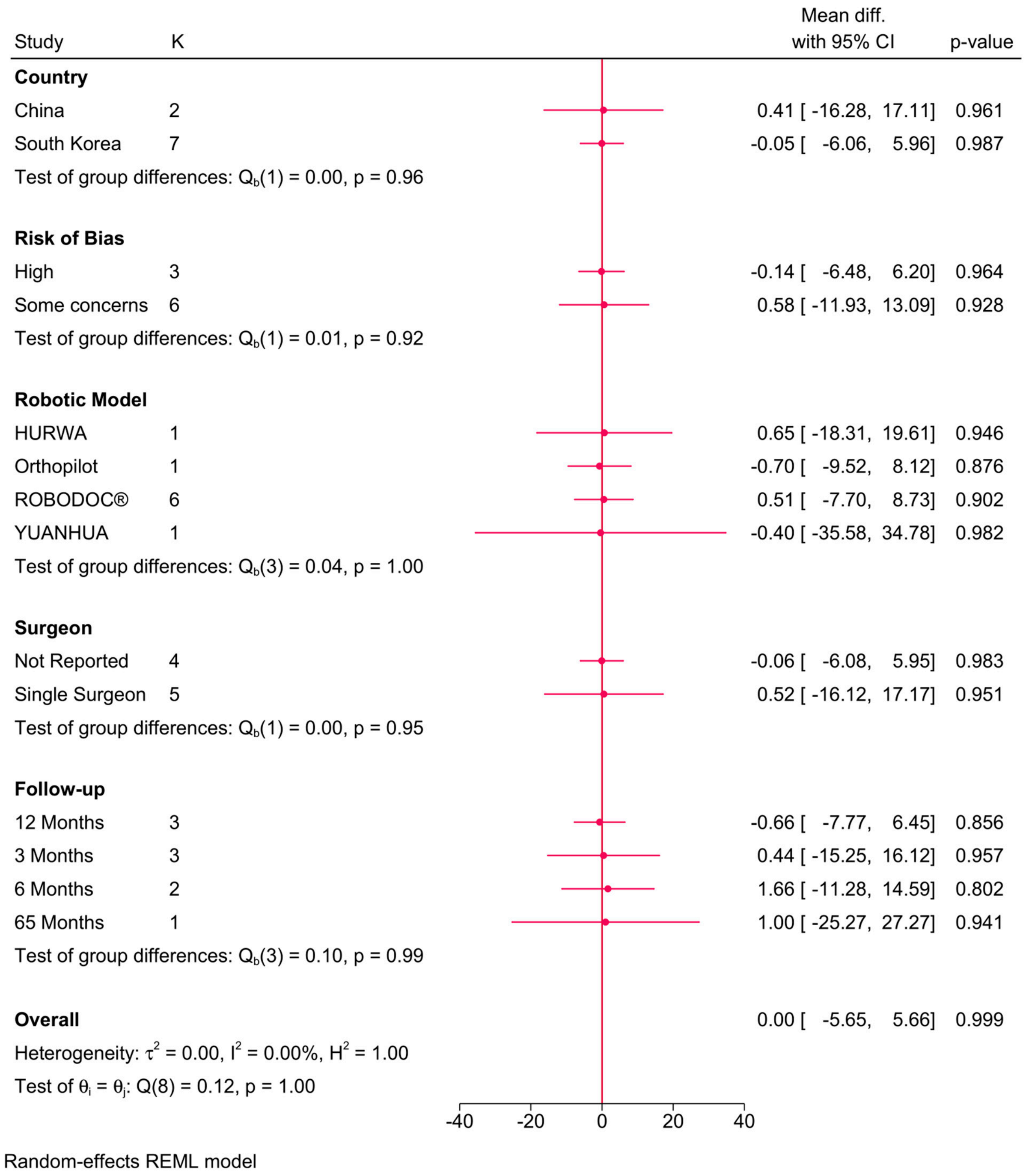

Figure S4. Subgroup analysis of the difference in HSS score between robot-assisted and conventional total knee arthroplasty based on country, risk of bias, robotic system, number of surgeons, and follow-up period

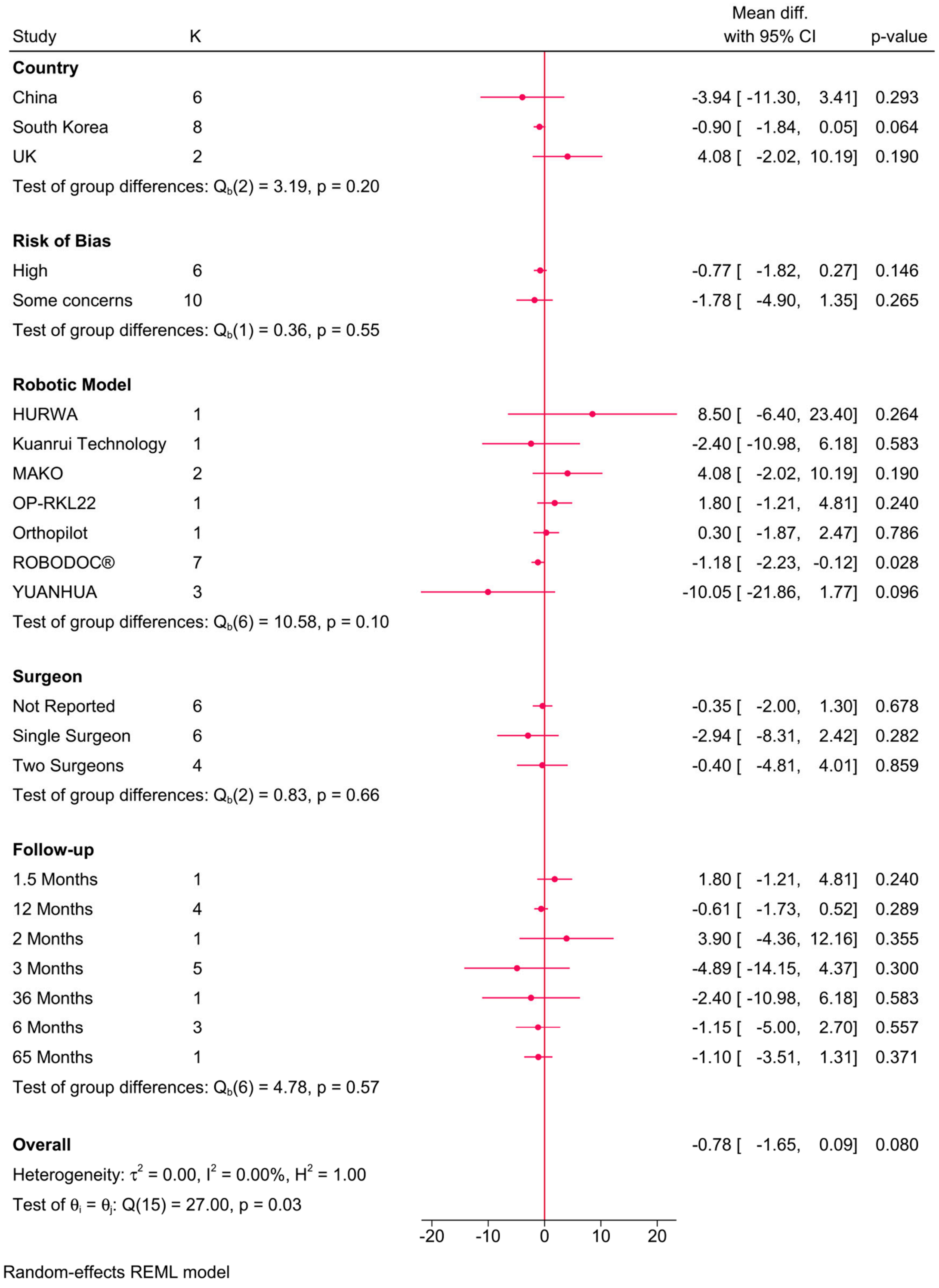

Figure S5. Subgroup analysis of the difference in WOMAC score between robot-assisted and conventional total knee arthroplasty based on country, risk of bias, robotic system, number of surgeons, and follow-up period

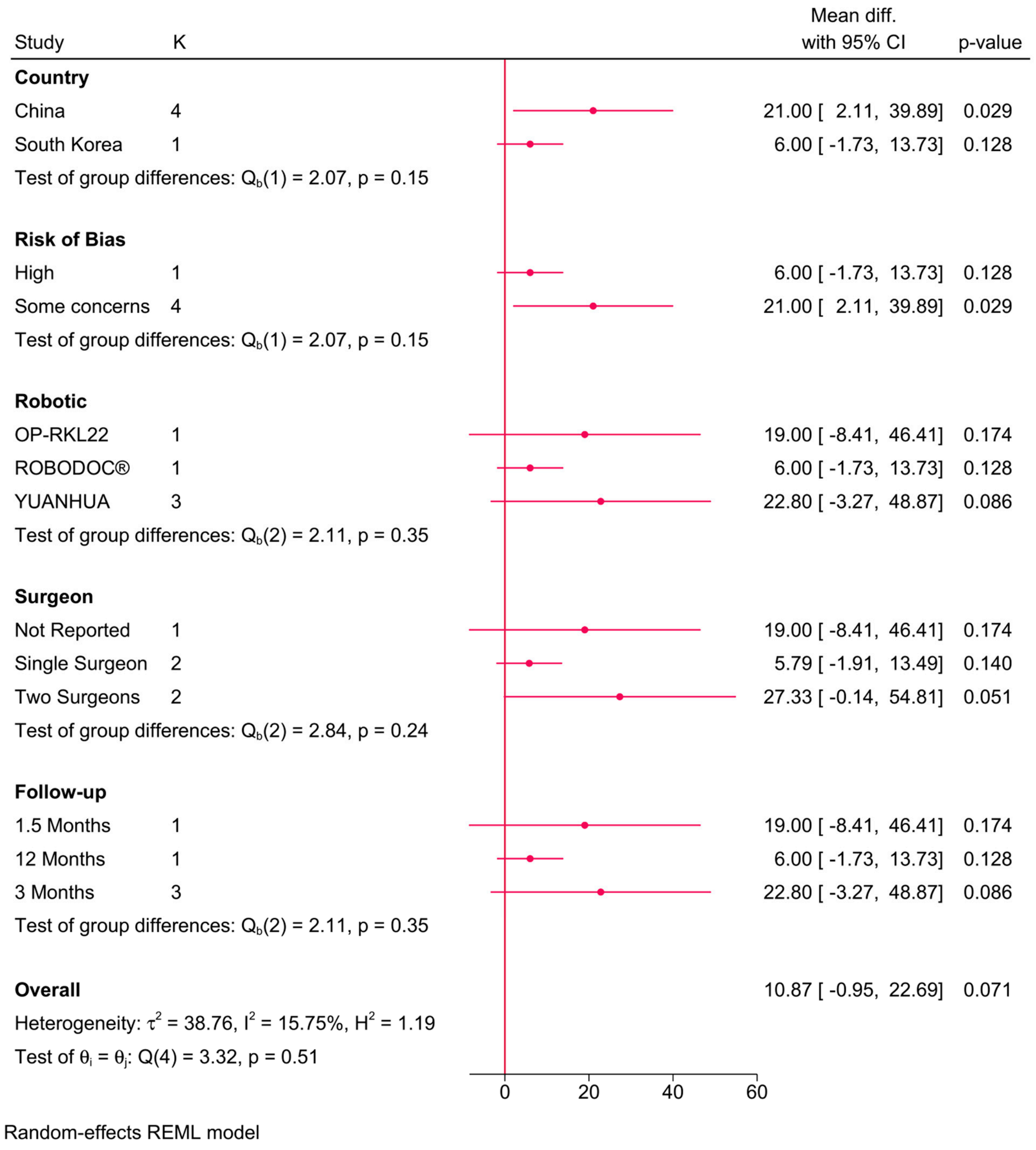

Figure S6. Subgroup analysis of the difference in intraoperative blood loss between robot-assisted and conventional total knee arthroplasty based on country, risk of bias, robotic system, number of surgeons, and follow-up period

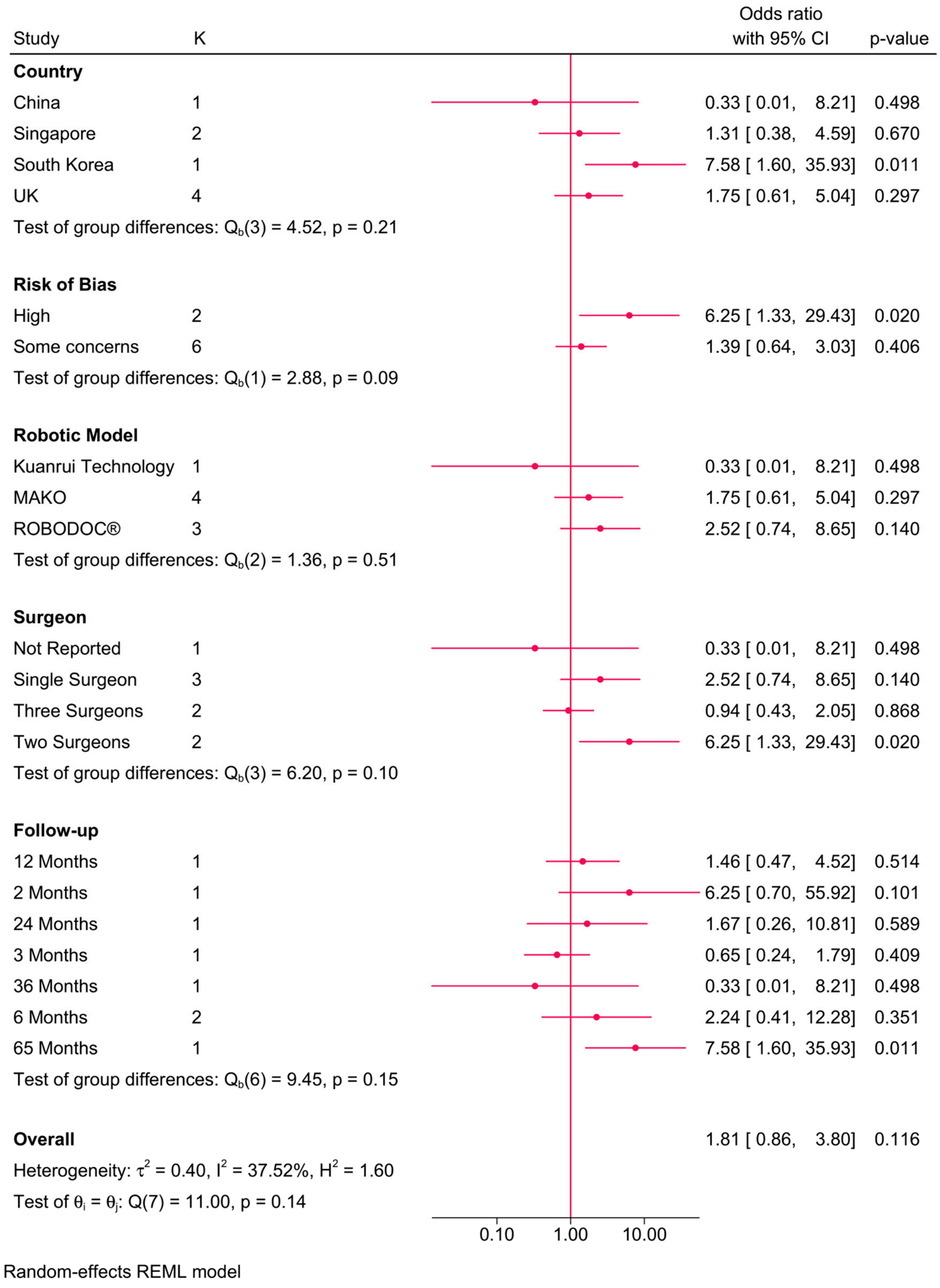

Figure S7. Subgroup analysis of the difference in satisfaction rate between robot-assisted and conventional total knee arthroplasty based on country, risk of bias, robotic system, number of surgeons, and follow-up period

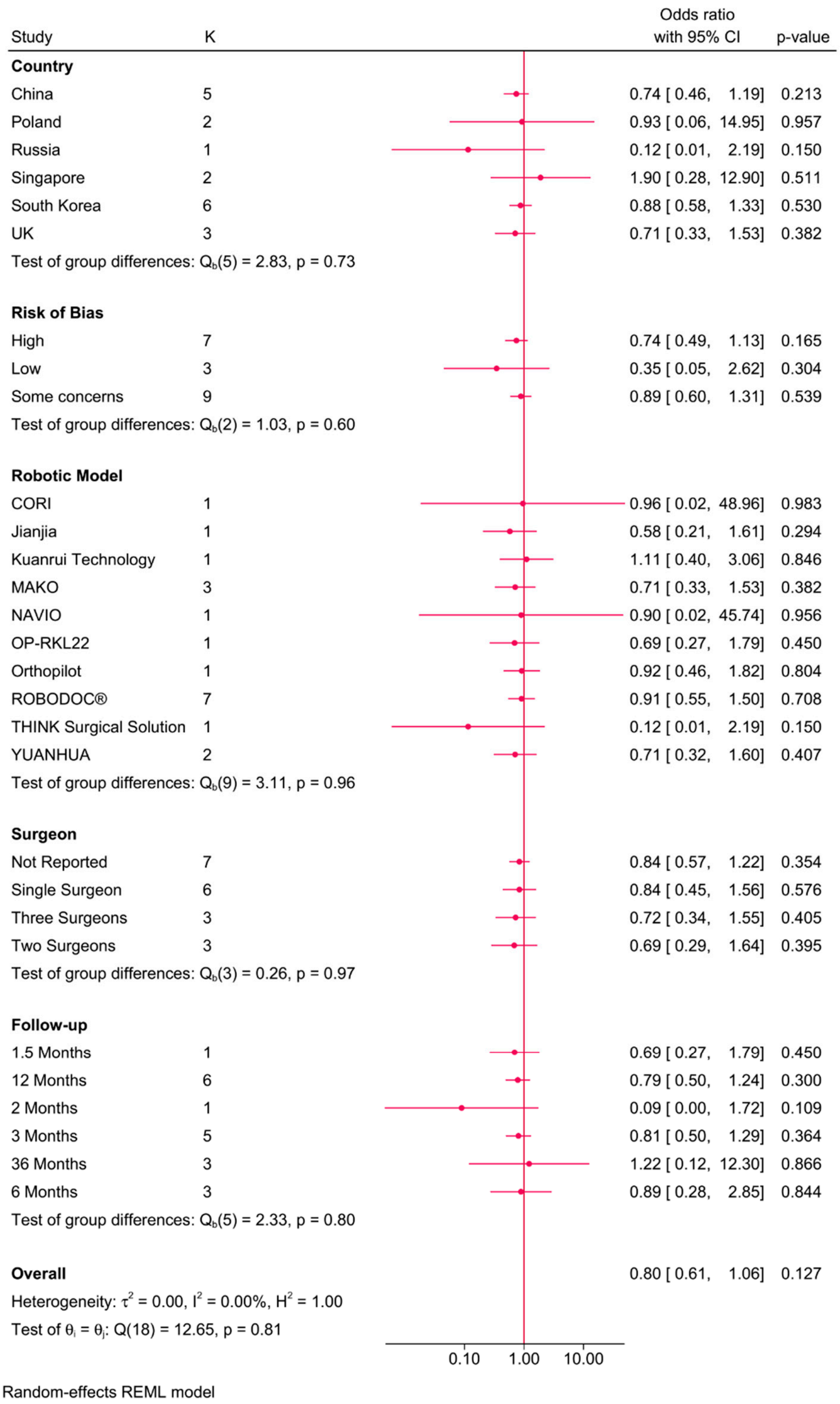

Figure S8. Subgroup analysis of the difference in overall complication rate between robot-assisted and conventional total knee arthroplasty based on country, risk of bias, robotic system, number of surgeons, and follow-up period

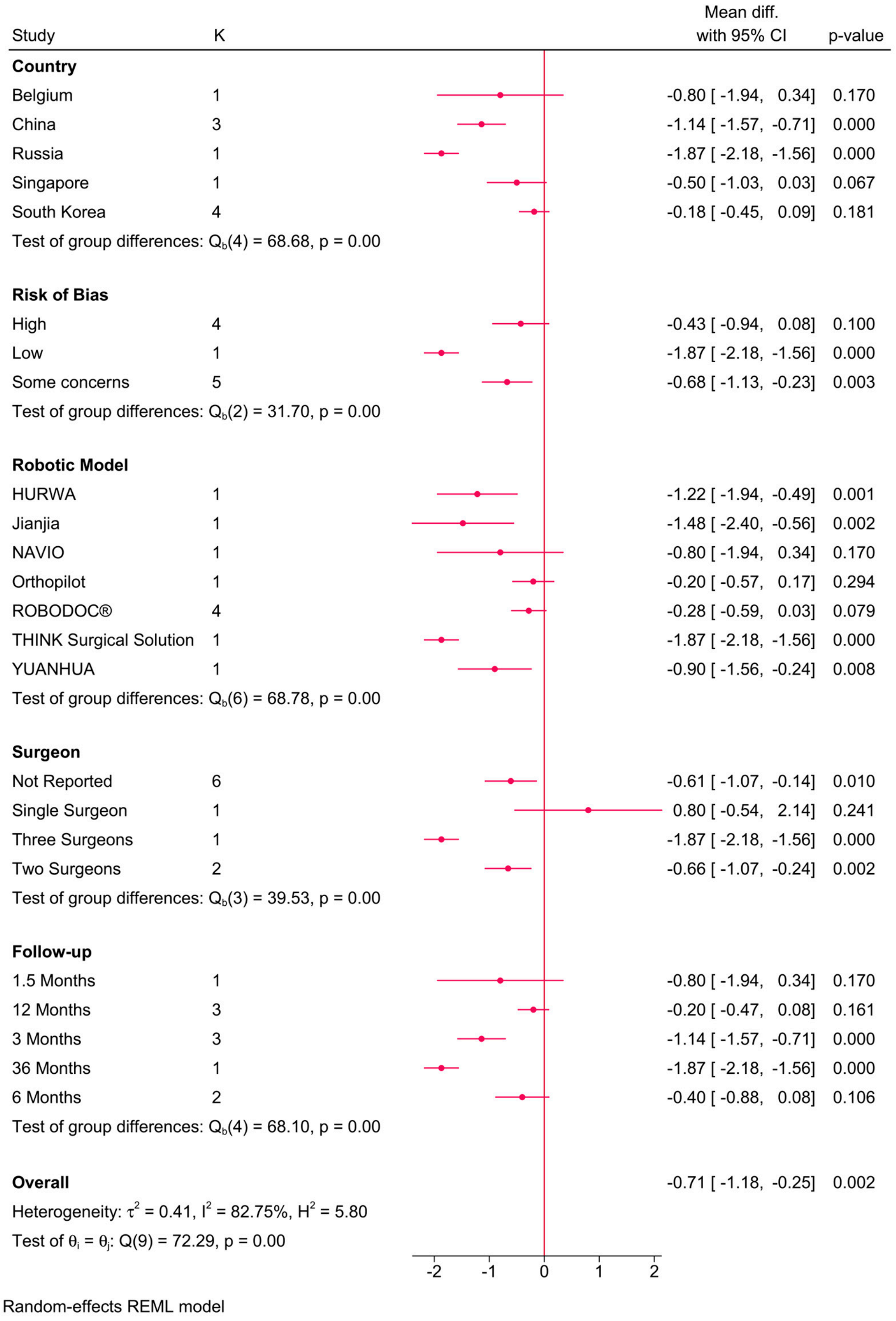

Figure S9. Subgroup analysis of the difference in HKA deviation between robot-assisted and conventional total knee arthroplasty based on country, risk of bias, robotic system, number of surgeons, and follow-up period

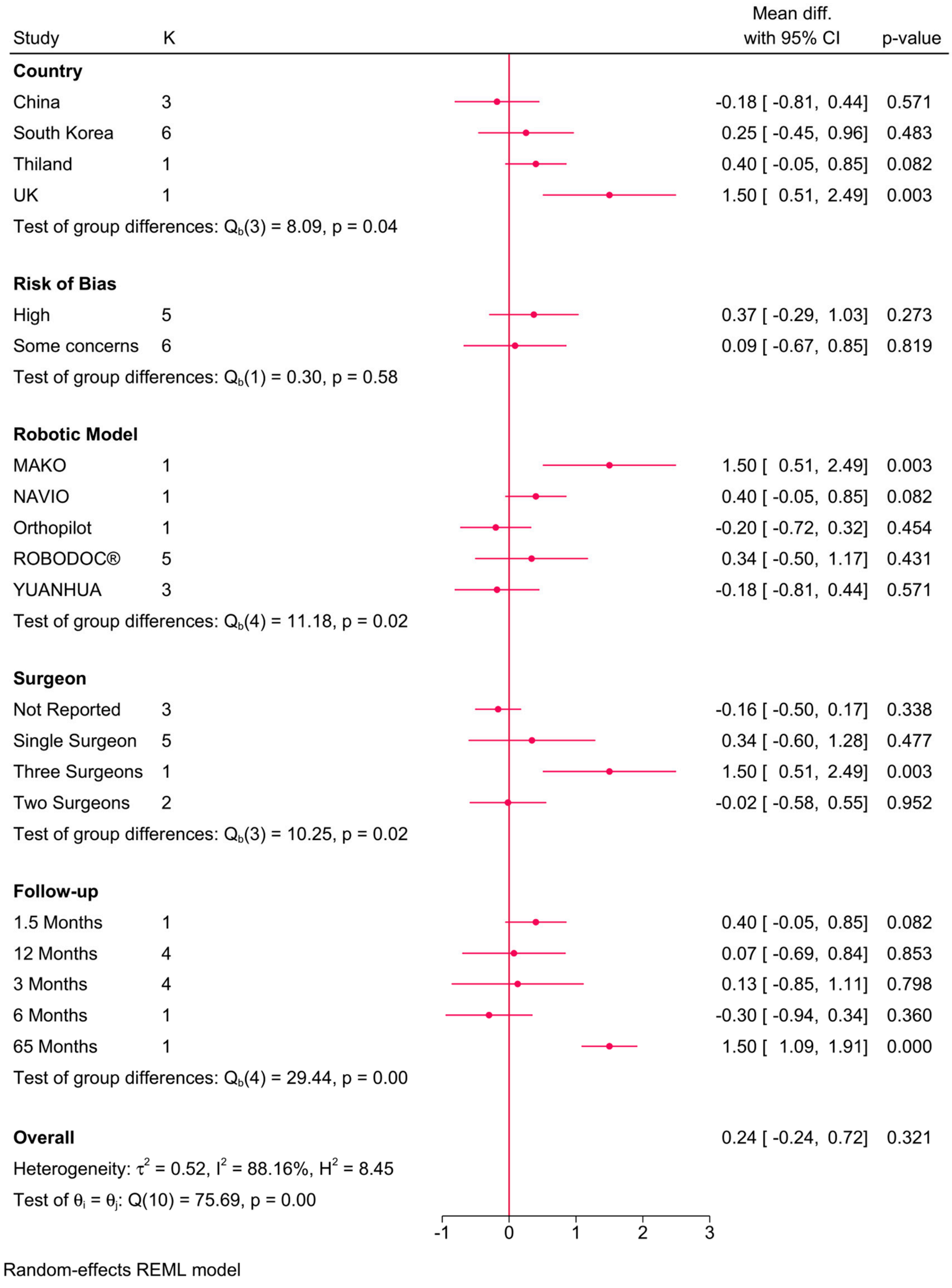

Figure S10. Subgroup analysis of the difference in FCIA between robot-assisted and conventional total knee arthroplasty based on country, risk of bias, robotic system, number of surgeons, and follow-up period.

FCIA: Femoral Coronal Inclination Angle

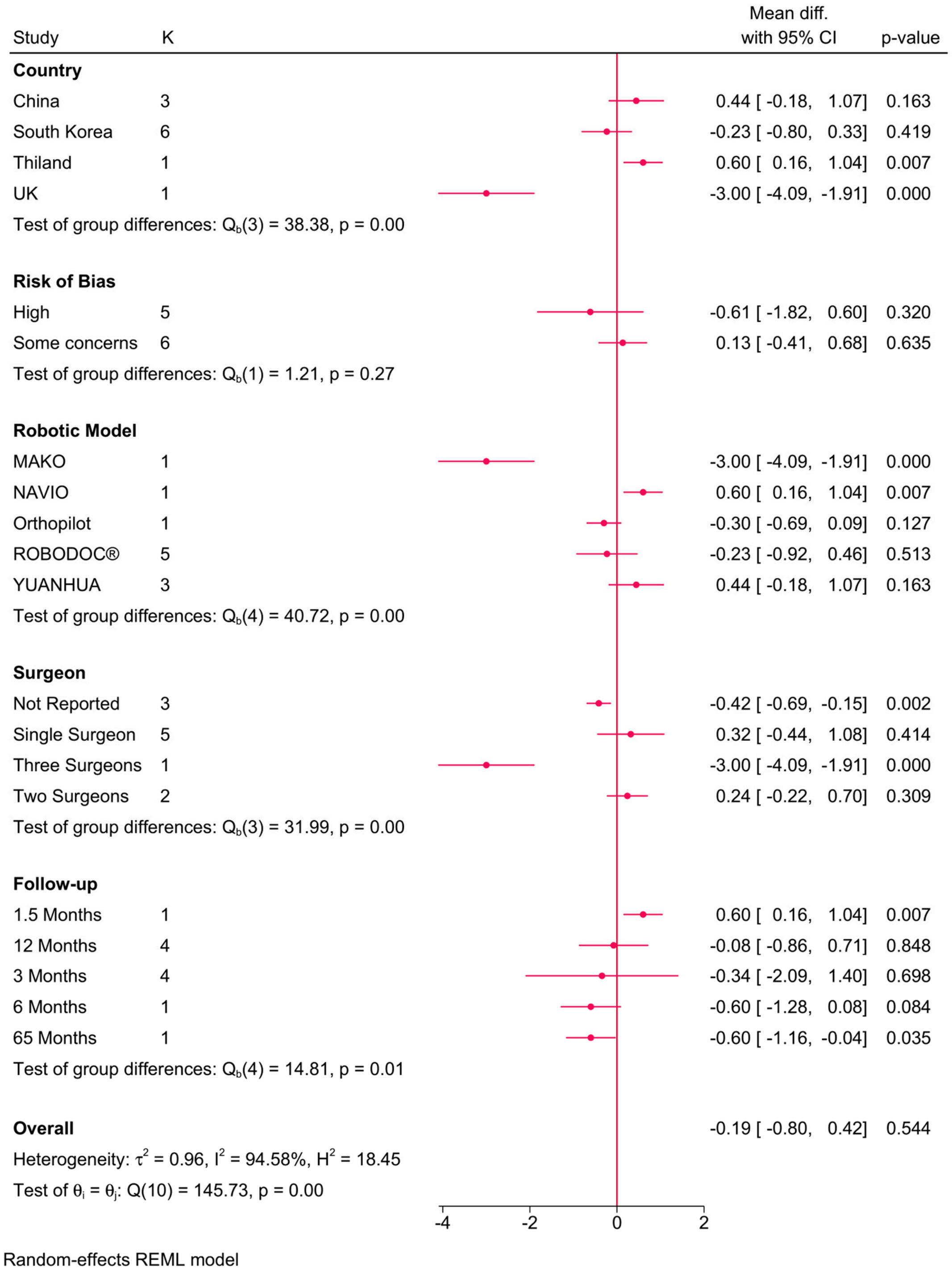

Figure S11. Subgroup analysis of the difference in TCIA between robot-assisted and conventional total knee arthroplasty based on country, risk of bias, robotic system, number of surgeons, and follow-up period. TCIA: Tibial Coronal Inclination Angle

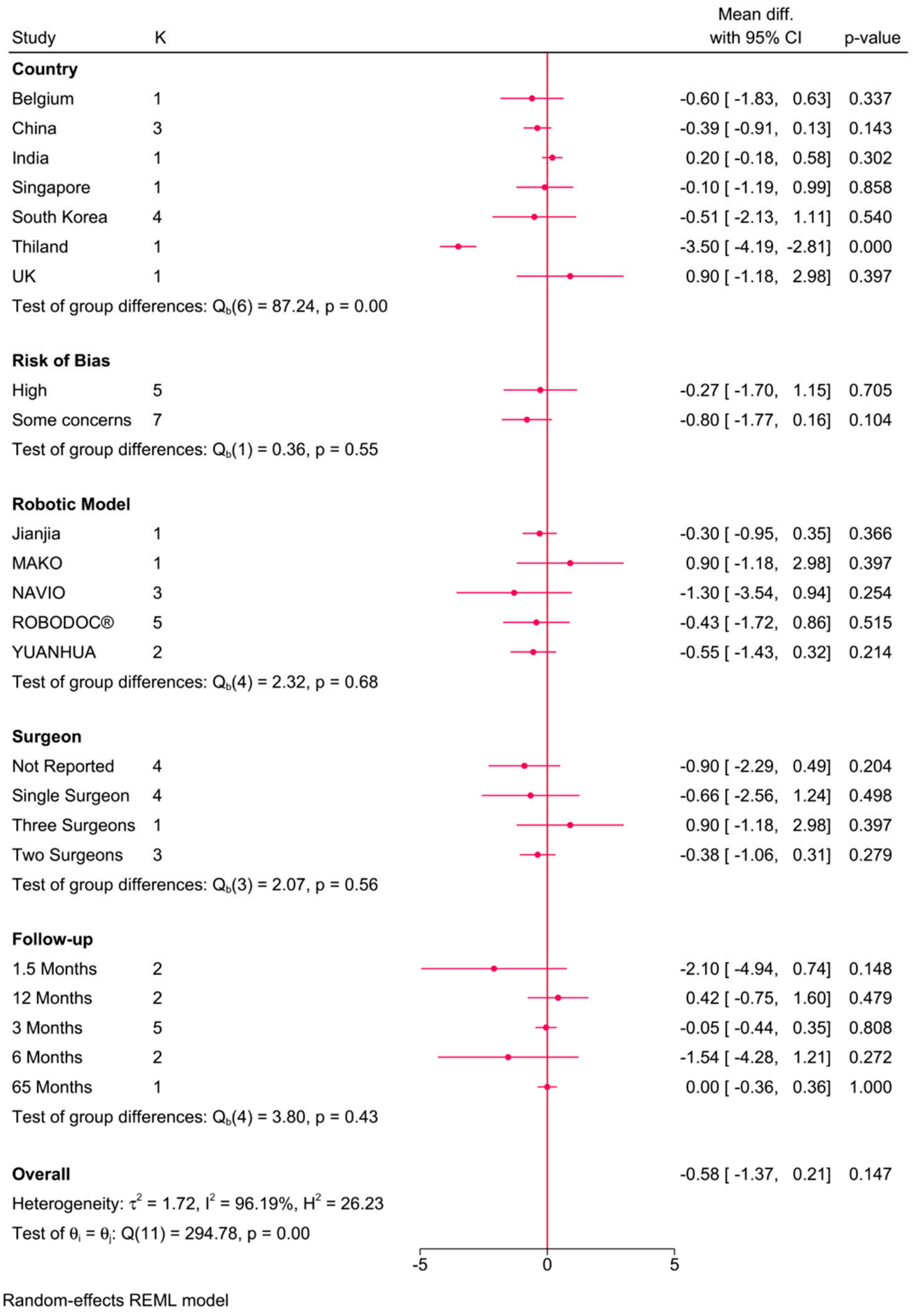

Figure S12. Subgroup analysis of the difference in FSIA between robot-assisted and conventional total knee arthroplasty based on country, risk of bias, robotic system, number of surgeons, and follow-up period. FSIA: Femoral Sagittal Inclination Angle.

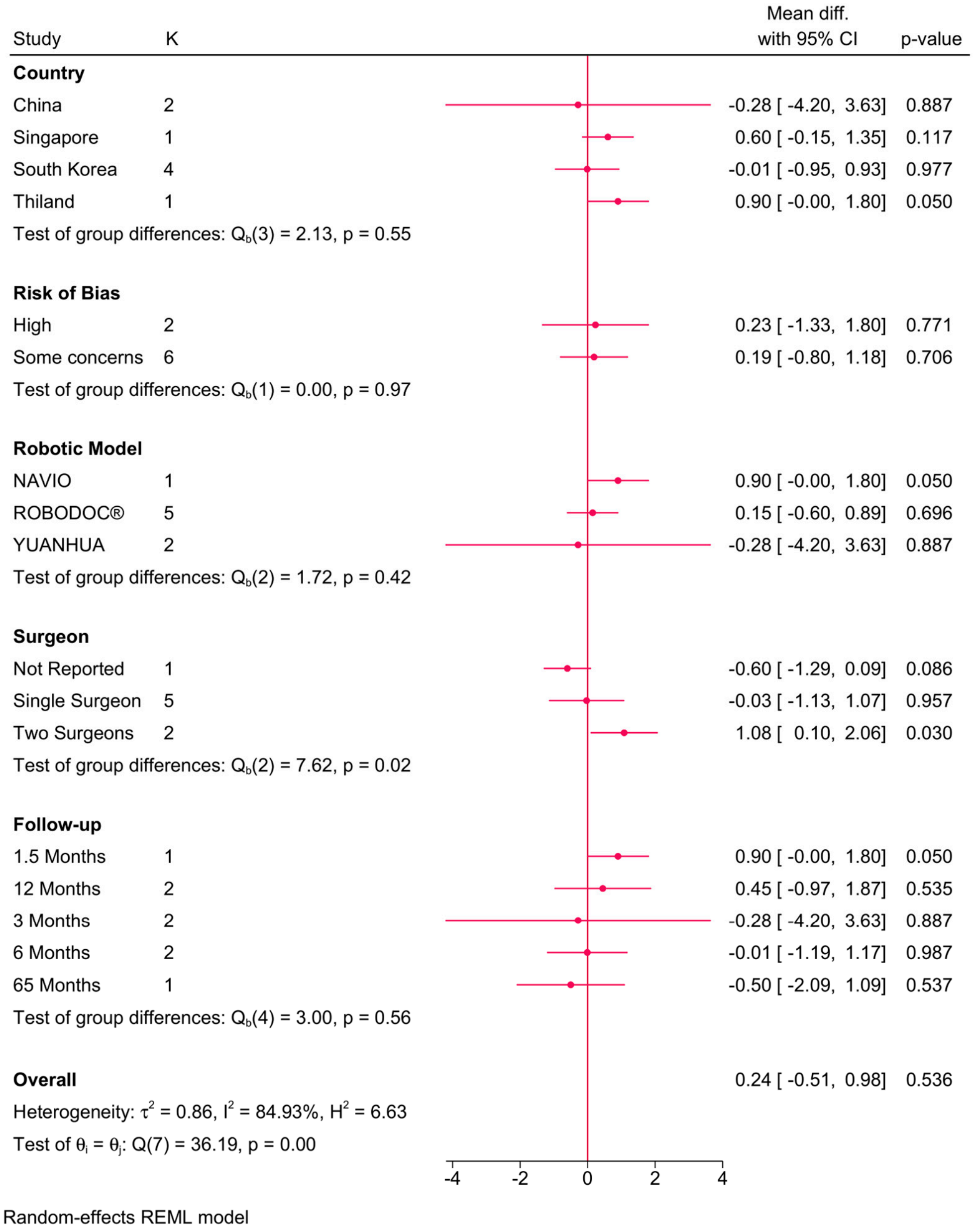

Figure S13. Subgroup analysis of the difference in TSIA between robot-assisted and conventional total knee arthroplasty based on country, risk of bias, robotic system, number of surgeons, and follow-up period. TSIA: Tibial Sagittal Inclination Angle.

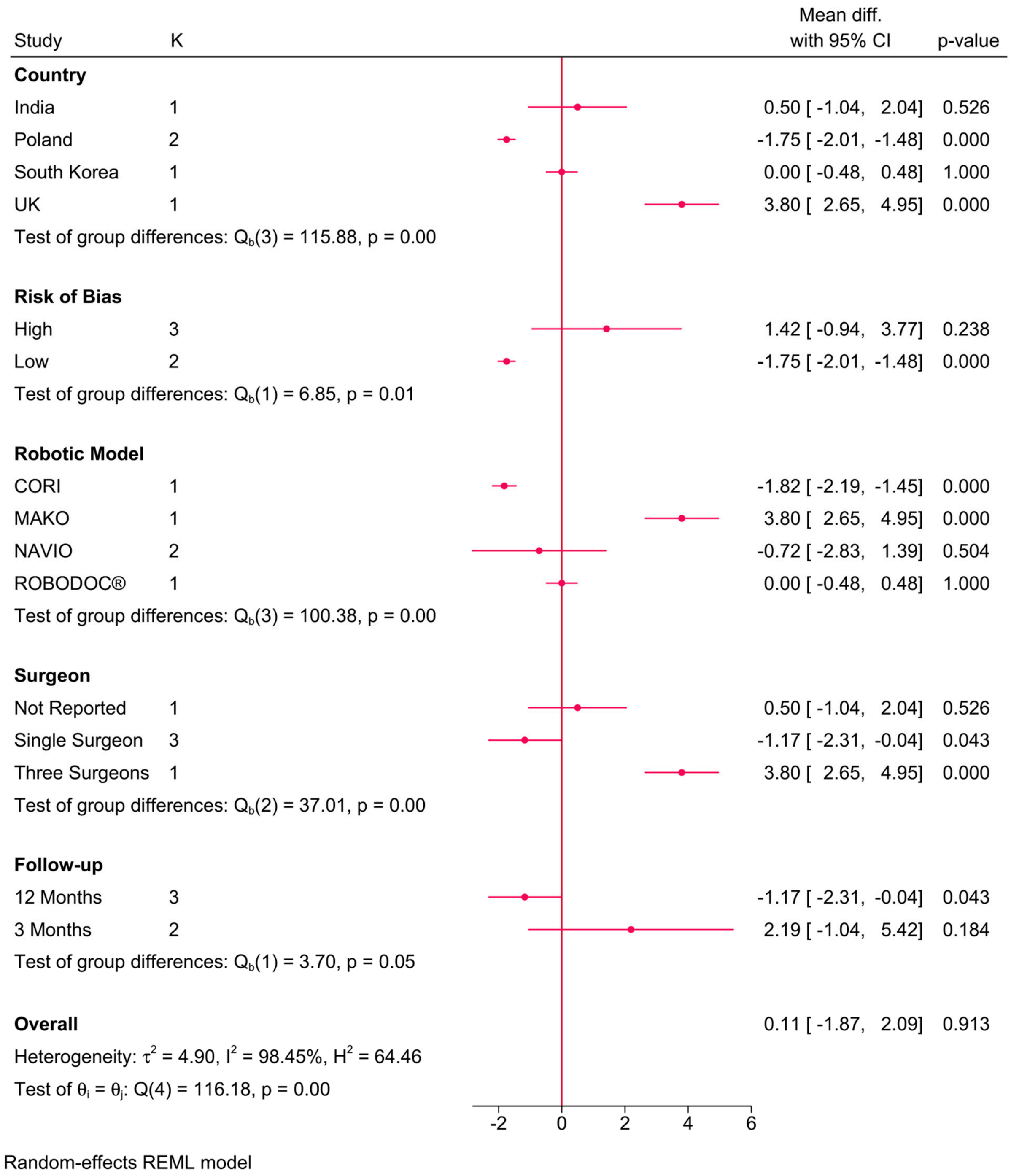

Figure S14. Subgroup analysis of the difference in TFA between robot-assisted and conventional total knee arthroplasty based on country, risk of bias, robotic system, number of surgeons, and follow-up period. TFA: Transverse Femoral Angle

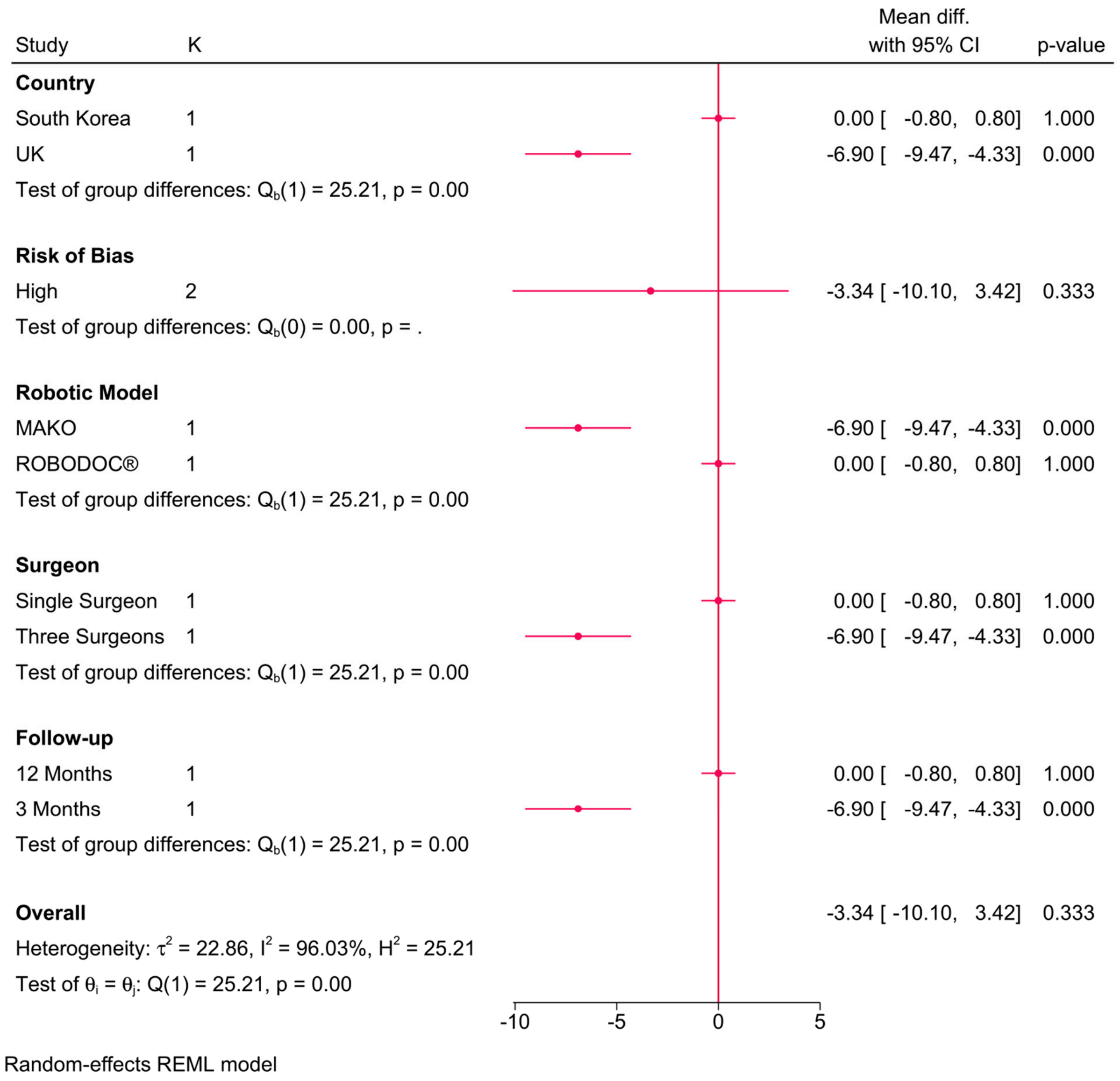

Figure S15. Subgroup analysis of the difference in TTA between robot-assisted and conventional total knee arthroplasty based on country, risk of bias, robotic system, number of surgeons, and follow-up period. TTA: Transverse Tibial Angle.
