# Supplementary material for: Comparative Efficacy and Precision of Robot-Assisted vs. Conventional Total Knee Arthroplasty: A Systematic Review and Meta-Analysis of Randomized Controlled Trials
Source: J Clin Med. 2025 May 7;14(9):3249. doi: 10.3390/jcm14093249 (PMC12072899; doi:10.3390/jcm14093249)
Supplement: Supplementary file 1 [file jcm-14-03249-s001.zip › Supplementary File S1.pdf]

Supplementary File S1

Radiographic Outcomes

The findings of radiographic outcomes are presented in **Supplementary File S1**.

1. HKA Angle

The HKA angle was significantly influenced by country (p=0.01), risk of bias (p=0.00), robotic system (p=0.01), and the number of surgeons (p=0.03) (**Figure 8**). RA-TKA demonstrated a significantly higher HKA angle than C-TKA in China and Thailand, particularly in RCTs with some concerns, and with the NAVIO (MD=0.50; 95%CI: 0.17, 0.83) and YUANHUA (MD=1.04; 95%CI: 0.46, 1.62) robotic systems at six weeks of follow-up. However, RA-TKA was associated with a lower HKA angle in the UK, high-risk RCTs, and procedures using the MAKO system (MD=-1.70; 95%CI: -3.25, -0.15) or performed by three surgeons. Meta-regression identified the robotic system as the only significant predictor, with the MAKO system showing a threefold lower HKA angle than ROBODOC (coefficient=-3.00, p=0.001) (**Table 2**).

2. HKA Deviation

All examined covariates significantly modified the effect on HKA deviation (**Figure S9**). RA-TKA was associated with a significantly lower deviation than C-TKA in China, Russia, and low-risk RCTs. A similar reduction was observed with the HURWA, Jianjia, THINK Surgical Solution, and YUANHUA robotic systems, whereas no difference was found with Orthopilot and ROBODOC. The reduction favoring RA-TKA was noted only at three and 36 months postoperatively, with no significant predictors identified in meta-regression (**Table 2**).

3. Femoral Coronal Inclination Angle (FCIA)

The robotic system (p=0.02) and country (p=0.04) significantly influenced FCIA (**Figure S10**). No significant differences between RA-TKA and C-TKA were found in most comparisons, except in the UK,

where the MAKO system resulted in superior FCIA (MD=1.50; 95%CI: 0.51, 2.49) at 65 months postoperatively. Meta-regression confirmed that the robotic system was the only significant determinant, with YUANHUA associated with a lower FCIA compared to ROBODOC (coefficient=-6.00, p=0.041) (**Table 2**).

4. Tibial Coronal Inclination Angle (TCIA)

The robotic system (p=0.00), country (p=0.00), number of surgeons (p=0.00), and follow-up (p=0.01) were significant effect-modifiers of TCIA (**Figure S11**). RA-TKA resulted in significantly lower TCIA compared to C-TKA in the UK with the use of the MAKO system (MD=-3.00; 95%CI: -4.09, -1.91).

5. Femoral Sagittal Inclination Angle (FSIA)

Country was the only significant effect-modifier of FSIA (**Figure S12**). No differences in FSIA were noted between RA-TKA and C-TKA except for Thailand (MD=-3.50; 95%CI: -4.19, -2.81).

6. Tibial Sagittal Inclination Angle (TSIA)

The number of surgeons was the only significant effect-modifier of TSIA (**Figure S13**). No differences in TSIA were noted between RA-TKA and C-TKA except for RCTs with 2 performing surgeons (MD=1.08; 95%CI: 0.10, 2.06). The meta-regression analysis showed that the robotic system was the only determinant of TSIA, with NAVIO (coefficient=1.67, p=0.000) and CORI (coefficient=-1.82, p=0.000) systems being associated with lower TSIA compared to ROBODOC, while MAKO system had higher TSIA (coefficient=3.80, p=0.000) (**Table 2**).

7. Transverse Femoral Angle (TFA)

All examined covariates significantly influenced TFA (**Figure S14**). RA-TKA resulted in a higher TFA in the UK when performed with the MAKO system (MD=3.80; 95%CI: 2.65, 4.95), whereas a lower TFA was observed in Poland, in low-risk RCTs, and when using the CORI robotic system (MD=-1.82; 95%CI: -2.19, -1.45), as well as at 12 months follow-up.

8. Transverse Tibial Angle (TTA)

All examined covariates significantly influenced TTA (**Figure S15**). However, due to the limited sample size (two RCTs), no meaningful conclusions could be drawn from this analysis.
